# Membranes for bioethanol production by pervaporation

**DOI:** 10.1186/s13068-020-01857-y

**Published:** 2021-01-07

**Authors:** Ping Peng, Yongqiang Lan, Lun Liang, Kemeng Jia

**Affiliations:** 1 Laboratory of Membrane Science and Technology, School of Resource and Chemical Engineering, Sanming University, Sanming, 365004 Fujian China; 2 Key Laboratory of Biobased Material Science & Technology (Education Ministry), Northeast Forestry University, Harbin, 150040 China

**Keywords:** Pervaporation, Membrane, Ethanol, Polymer

## Abstract

**Background:**

Bioethanol as a renewable energy resource plays an important role in alleviating energy crisis and environmental protection. Pervaporation has achieved increasing attention because of its potential to be a useful way to separate ethanol from the biomass fermentation process.

**Results:**

This overview of ethanol separation via pervaporation primarily concentrates on transport mechanisms, fabrication methods, and membrane materials. The research and development of polymeric, inorganic, and mixed matrix membranes are reviewed from the perspective of membrane materials as well as modification methods. The recovery performance of the existing pervaporation membranes for ethanol solutions is compared, and the approaches to further improve the pervaporation performance are also discussed.

**Conclusions:**

Overall, exploring the possibility and limitation of the separation performance of PV membranes for ethanol extraction is a long-standing topic. Collectively, the quest is to break the trade-off between membrane permeability and selectivity. Based on the facilitated transport mechanism, further exploration of ethanol-selective membranes may focus on constructing a well-designed microstructure, providing active sites for facilitating the fast transport of ethanol molecules, hence achieving both high selectivity and permeability simultaneously. Finally, it is expected that more and more successful research could be realized into commercial products and this separation process will be deployed in industrial practices in the near future.

**Graphical abstract:**

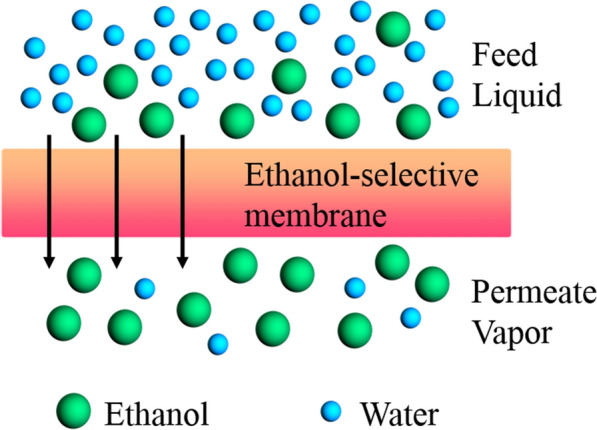

## Background

In the International Energy Outlook 2016 and 2019 (IEO2016 and IEO2019) Reference cases, total world energy consumption rises from 549 quadrillion British thermal units (Btu) in 2012 to 911 quadrillion Btu in 2050, an increase of nearly 66% [1, 2]. Renewable energy is the world’s fastest growing form of energy source, and its consumption increases by 3% per year between 2018 and 2050. Nuclear energy consumption grows by 1% per year [2]. However, more than three-fourths of the worldwide consumed energy in 2040 is still supplied by non-renewable fossil energy sources (coal, natural gas, crude oil/petroleum, etc.) [1]. Over consumption of fossil fuels leads to the deterioration of the ecological environment, such as acid rain, and global warming. [3]. Alternative renewable energy sources have been drawing more and more attention throughout the world in terms of environmental friendliness and economic viability [4]. Water, biomass, wind, and geothermal heat have the potential application as a viable substitute for traditional fossil fuels [5]. Biomass-based fuels cause widespread concern around the world because biofuels are renewable, sustainable, commonly available, environmentally benign, and biodegradable [6].

Biofuels include bioethanol, biobutanol, biomethanol, biodiesel, bio-oil, biogas, biohydrogen and so on. To date, bioethanol and bioethanol–gasoline blends are the most commonly used vehicle fuels and are considered as promising alternatives to conventional petroleum [7, 8]. Theoretically, any of alcohols including methanol, ethanol, propanol, and butanol can be used for petrol engines due to their oxygen enrichment, octane enhancer, and reduction of carbon monoxide and unburned hydrocarbons emissions that increases engine efficiency and performance [9]. Only methanol and ethanol fuels, however, are economically and technically feasible for internal combustion engines. Unfortunately, methanol has toxicity. The use of bioethanol–gasoline-blended fuels for automobiles is beneficial to not only greatly reducing the consumption of petroleum but also the emissions of greenhouse gases such as CO_2_, CO, SO_2_, HC, and NO_x_ [8, 10]. From the viewpoint of the life cycle assessment, bioethanol produced from corn through the fermentation process raises the energy by 20–30% than that of fossil fuel used to produce it, while bioethanol produced from cellulosic and sugarcane yields nine times as much energy as fossil energy consumed [7]. Cellulose is the most abundant natural plant resource in the world, so the production of fuel ethanol by fermenting cellulose has become a guarantee of sustainable development [11]. Unfortunately, fermentation broths typically contain ethanol at less than 10 wt. % since a higher concentration of ethanol would have a suppression effect on microorganisms for bioethanol fermentation, thereby leading to the stop of the fermentation process [12]. Therefore, an important step in the cellulose-to-ethanol conversion process is the extraction of ethanol from fermentation broths. A conventional separation process is intermittent, at first, a batch of fermenting liquid is distilled [13], and then the obtained azeotrope products (95.6 wt. % ethanol) are separated and dehydrated to meet fuel specifications. Distillation requires a tremendous amount of energy, meaning high capital costs [14]. Apparently, such intermittent production process is low-efficiency and high-energy consumption, which does not accord with requirements of sustainable development and circular economy.

Pervaporation (PV), as an emerging membrane separation technology, has received increasing attention as Kober first proposed the concept of PV at the beginning of the last century [15]. Nowadays, PV has broadly enlisted by researchers for the separation and purification of biofuels by means of its advantages including highly efficient separation, simple equipment, low cost, low pollution, and low-energy consumption since the separation of the liquid mixture achieved by PV refers to the difference in the dissolution rate [16]. The PV in conjunction with a fermentor cannot only achieve continuous biomass-ethanol production but also reduce the energy, economic, and environmental costs. This article attempts to provide a review on the research progress in ethanol-selective PV membranes from the perspectives of transport mechanisms, common fabrication methods, and membrane materials. It will benefit a lot for taking inspiration from the previous works, and finding new ideas and strategies for developing a new generation of high-performance and high-stability PV membranes for ethanol recovery.

## Transport mechanisms of PV

In the PV process, the desired component in feed preferentially permeates through a membrane, evaporates into vapor, and enriches at the permeate side of the membrane [17]. Due to the complex interaction between membrane materials and permeating components, the mass transfer in the membranes is considerably complicated. So far, there is no such a universal model to characterize every detail of mass transfer in membranes [18]. The permeation flux is significantly dependent on the physical chemistry properties of the membranes, such as thickness [19], affinity for permeate components [20, 21], and diffusion coefficient of permeate components across membranes [22, 23]. For example, the permeation flux is inversely proportional to the membrane thickness. This is principally because that the thinner the membrane is, the lower the overall mass transfer resistance of permeation components through the membrane, therefore, the greater the permeation flux [24]. To prepare membranes with high-PV performance, it is critical to understand the transport mechanism [25].

### Facilitated transport mechanism

The facilitated transport mechanism is proposed to explain the preferential permeation of a species (either gas or liquid) through a mixed matrix membrane. Fillers in the membrane act as facilitated transport carriers, to accelerate the transfer of desired species. According to mobility, they can be classified into three categories: free-moving carriers, semi-mobile carriers, and fixed-site carriers [26]. In most cases, nanoparticle-filled membranes fall into the last category due to they are covalently trapped into matrices. In this situation for ethanol PV separation, a reversible chemical reaction (C + E ⇔ CE) occurs between each carrier (C) and ethanol molecule (E) in the membrane, whereby ethanol preferentially diffuses through the membrane, whereas C and CE only exist in the inside of the membrane due to the mobile constraint of C. This is the reason that the hybrid membranes are selective for ethanol. At present, almost only the dual-mode sorption model and resistor–capacitor (RC) circuit model are put forward to systematically unravel this facilitated transport mechanism for fixed-site hybrid membranes.

The dual-mode sorption models for gas transport were based on three hypotheses. First, it was hypothesized that a membrane should consist of two clearly different regions that one obeyed the Henry’s law with gas solubility and the other followed a Langmuir sorption isotherm. Second, both regions were assumed to be in equilibrium. Third, it was further assumed that the transport process was diffusion limited. It was also indicated that only when fixed-site carriers reached a certain concentration, species could directly transfer between two the carriers [27].

Up to now, based on the traditional theory, some new progress has been made, such as the appropriate expression for the “effective” diffusion coefficient [28], the theory of chained carrier for facilitated diffusion in solid membranes [29], and the model for facilitated mass transport with fixed-site carrier membranes [30]. However, these models are confined by the elementary reversible reaction (C + E ⇔ CE) that is simplified to make theoretical analysis easy, leading to their inability to fully explain many complicated practical situations [31].

### Solution–diffusion mechanism

**Fig. 1:**
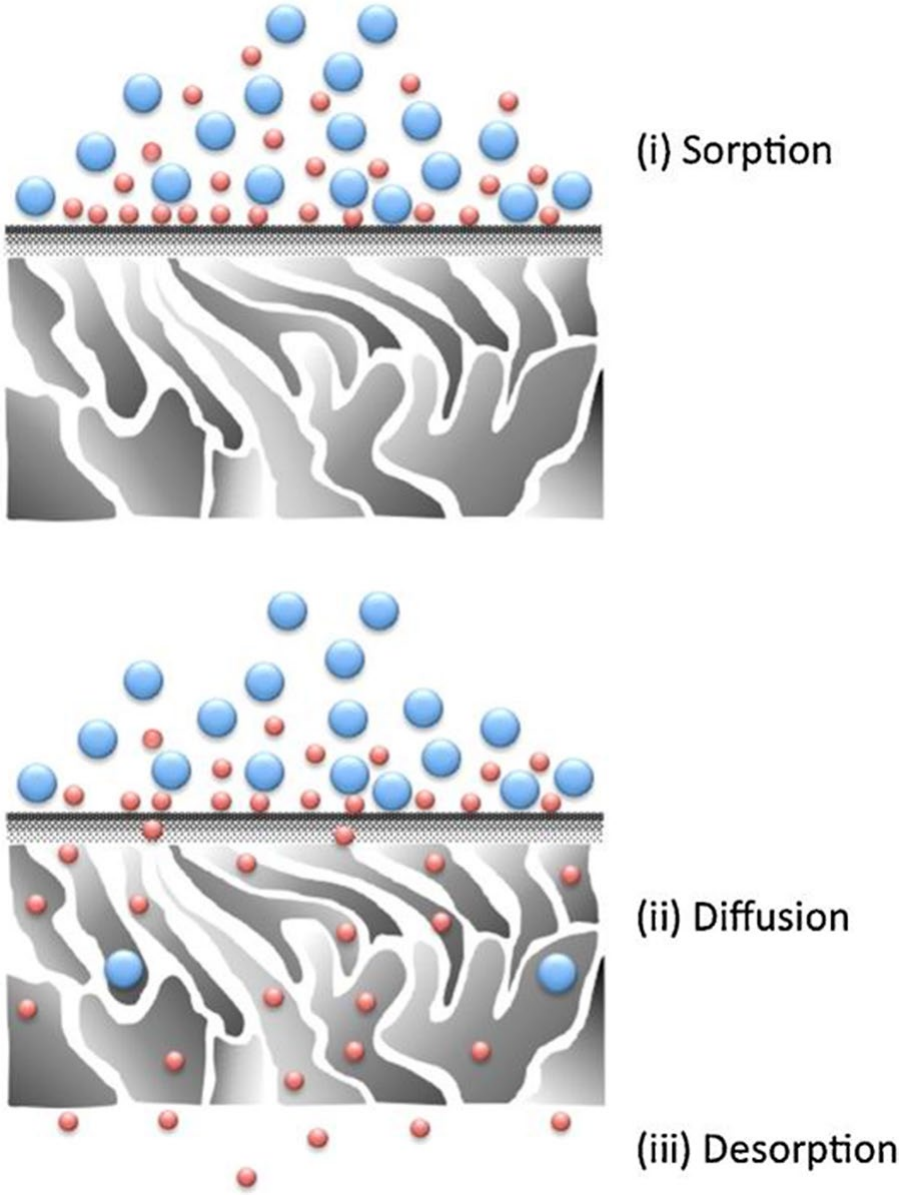
Schematic of the solution–diffusion mechanism [32]

The solution–diffusion mechanism, first developed by Thomas Graham to elucidate gas penetrant across membranes, is the currently most widely accepted transport mechanism. Up to now, it is extended to other separations technologies, such as PV, nanofiltration, reverse osmosis as well as dialysis. According to the solution–diffusion model, the transport of permeating species through a membrane involves three distinct and consecutive steps: solution (also known as sorption), diffusion, and desorption, as pictorially shown in Fig. 1 [32]. When reaching a solution equilibrium, the overall rate is determined by the first two steps since molecules desorb generally with extreme rapidity. Therefore, the activity coefficient in the solution process can be theoretically calculated based on the Henry’s Law and Flory–Huggins theory in which the simplifying assumptions are no and intense interactions between permeating species and membrane material, corresponding to two extreme cases. In contrast, the diffusion process is an irregular diffusion, which is not completely governed by the Fick’s first law [33]. In light of this situation, Fujita developed the free volume theory to illustrate the diffusion behavior of permeates in polymeric membranes. The permeating molecules were assumed to diffuse essentially along with the polymeric interchain spacing and internal micropores of the membranes [34]. Currently, this theory is generally applicable to explain the diffusion of penetrants through mixed matrix membranes.

From the solution–diffusion mechanism standpoint, the PV performance of membranes would be enhanced by elevating either solubility selectivity or diffusivity selectivity. The former is thermodynamically controlled, whereas the latter is a kinetically favorable process. The membrane solubility selectivity favors more condensable molecules or molecules with special interactions with membrane materials [35]. In practice, this interaction is usually assessed by means of the Hildebrand and/or Hanson solubility parameter [36]. Typically, the closer the solubility parameters of permeation component and membrane material, the higher the perm-selectivity of the permeation component would be [37]. The membrane diffusivity selectivity is primarily associated with properties of permeation components (i.e., shape and size) and membrane materials (i.e., flexibility and interchain spacing), as well as the intermolecular interaction between the permeating components themselves and the interaction between the permeating components and the membrane material. Therefore, it is possible to determine whether the membrane is water-selective or organic-selective by the choice of membrane material and the control of membrane morphology [38]. Interestingly, incorporation of appropriate inorganic materials or hybrids into polymer matrices is capable of consumedly improving both the solubility selectivity and the diffusivity selectivity of the hybrid membranes [39]. For ethanol perm-selective separation from water, it is confirmed that the addition of a hydrophobic material with a wide range of hydrophobic groups into membranes could improve both the solubility selectivity and the diffusivity selectivity. On the one hand, the hydrophobic fillers tend to provide a higher affinity of the membranes for ethanol rather than water, which can be responsible for the increase in solubility selectivity [40]. On the other hand, with regard to the diffusivity selectivity, it can be categorized into two cases: one is porous fillers; and the other is nonporous fillers. Porous fillers behave as molecular sieves attributing to their specific pore structure; and nonporous fillers create a good number of channels at the filler/matrix interface for ethanol to preferentially traverse the membranes, which results in the improvement of diffusivity selectivity. In addition, the incorporation of fillers is also capable of severely disrupting the inherent polymer chain packing, thereby improving the free volume inside the membranes, subsequently increasing the membrane permeability [41].

## Membrane performance

The PV separation performance of membranes is usually evaluated by the productivity and the separation ability for targeted components from mixtures that are expressed in terms of permeation flux and separation factor or permeability (or permeance) and selectivity, respectively [42]. In the case of a binary mixture, the permeation flux *J* and separation factor *β* can be experimentally obtained with the following equations as follows:

1 $$ J = \frac{Q}{A \cdot t}, $$

2 $$ \beta = \frac{{{{Y_{j} } \mathord{\left/ {\vphantom {{Y_{j} } {Y_{i} }}} \right. \kern-\nulldelimiterspace} {Y_{i} }}}}{{{{X_{j} } \mathord{\left/ {\vphantom {{X_{j} } {X_{i} }}} \right. \kern-\nulldelimiterspace} {X_{i} }}}}, $$

where *Q* stands for the total weight of permeates through the effective surface area *A* in time *t*. In addition, *Y* and *X* represent the mass fractions of species *i* and *j* on the permeate and feed sides, respectively.

The permeability *P*, permeance *P*/*l*, and selectivity *α* take into account the impact of the driving force. The permeation flux of species *i*, *J*_*i*_, is a strong function of its partial pressure gradient and inversely proportional to the membrane thickness *l* as follows:

3 $$ J_{i} = \frac{{P_{i} }}{l}\left( {p_{i,f} - p_{i,p} } \right) = \frac{{P_{i} }}{l}\left( {\gamma_{i} \chi_{i} p_{i}^{{{\text{sat}}}} - p_{i,p} } \right), $$

where *p*_*i,f*_ and *p*_*i,p*_ are the partial pressures of species *i* in feed and permeate vapor, respectively, whereas *γ*_*i*_ and *χ*_*i*_ denote the activity coefficient and the mass fraction of species *i* in the feed solution, respectively. In addition, *p*_*i*_^sat^ is the saturated vapor pressure of species *i*. Besides, the membrane selectivity for a binary mixture containing species *i* and *j*, *α*_*i,j*_, can be expressed as:

4 $$ \alpha_{i,j} = \frac{{P_{i} }}{{P_{j} }} = \frac{{D_{i} }}{{D_{j} }}\frac{{{{c_{{i,{\text{membr}}}} } \mathord{\left/ {\vphantom {{c_{{i,{\text{membr}}}} } {c_{{i,{\text{feed}}}} }}} \right. \kern-\nulldelimiterspace} {c_{{i,{\text{feed}}}} }}}}{{{{c_{{j,{\text{membr}}}} } \mathord{\left/ {\vphantom {{c_{{j,{\text{membr}}}} } {c_{{j,{\text{feed}}}} }}} \right. \kern-\nulldelimiterspace} {c_{{j,{\text{feed}}}} }}}}, $$

where *P*_*i*_ and *P*_*j*_ represent the aforementioned permeabilities of species *i* and *j*; *D*_*i*_ and *D*_*j*_ are the diffusion coefficients of species *i* and *j*; *c*_*i,*membr_ and *c*_*j,*membr_ are the concentrations of species *i* and *j* in the membrane surface; *c*_*i,*feed_ and *c*_*j,*feed_ are the concentrations of species *i* and *j* in the feed. *D* can be related to the *c*_membr_ of a particular species by the Fick’s first law as follows:

5 $$ J = - D\frac{{{\text{d}}c_{{{\text{membr}}}} }}{{{\text{d}}\delta }}, $$

where *δ* is the position variable. To decoupled the impact of “effective” membrane thickness, especially if the thickness of selective layer is unknown or the membrane is asymmetric, the selectivity *α*_*i,j*_ of species *i* and *j* can be further described as:

6 $$ \alpha_{i,j} = \frac{{{{P_{i} } \mathord{\left/ {\vphantom {{P_{i} } l}} \right. \kern-\nulldelimiterspace} l}}}{{{{P_{j} } \mathord{\left/ {\vphantom {{P_{j} } l}} \right. \kern-\nulldelimiterspace} l}}}. $$

## Fabrication of PV membranes

According to the membrane structure, PV membranes can be classified into dense membranes and asymmetric membranes. The dense membranes with the nonporous structure are relatively thick, commonly over 100 μm, and tend to deliver limited permeation flux since mass transport becomes limited, accordingly they are inappropriate for large-scale industrial PV separation. Alternatively, the asymmetric membranes are constructed of thin separation layers and micropores support layers. The porous support materials, such as polyvinylidene fluoride (PVDF) [43, 44] and cellulose acetate (CA) microfiltration membranes [45, 46], ceramic materials [23, 47], and other various customized porous materials [48–54], provide structural strength for fragile thin separation layers and enhance permeability without affecting the intrinsic selectivity because of the reduction of the effective thickness of separation layers. Membranes can be divided into three types in terms of their shapes: the flat-sheet [55–58], tubular [59–61], and hollow-fiber membranes [62, 63].

The flat-sheet membranes have been employed in bioreactor systems for recent decades [31]. In addition, most of the currently and commercially available membranes for recovering ethanol from water via PV are in the flat-sheet configuration on account of easy fabrication and convenient assembly [64–66]. Considering that the ethanol-selective membranes are almost polymer-based materials, only the fabrication of polymeric membranes (both dense and composite membranes) was discussed herein.

Dense flat-sheet membranes are frequently prepared by solution casting followed by solvent evaporation [32]. Typically, an artificial polymer is first and completely dissolved in an appropriate solvent. After degasification, the obtained dope solution is poured on a flat plate, allowed to spread, and subsequently left to evaporate the solvent [67]. Membranes with multiple selective layers, in sandwich-like structure, are constructed by a layer-by-layer solution casting method in much the same procedures as mentioned above [68]. Surface pre-treatment for a previously formed layer, if necessary, is applied prior to the coating of the next one to enhance the interlayer interaction [69]. In another case, the polymeric solutions can be coated on various porous supports consisting of either organic or inorganic substances to fabricate composite membranes [23, 45, 70]. Apart from the regular flat plates, the supports could be in hollow fiber or tubular format [50, 63, 71]. To prevent polymer solutions from penetrating and blocking the micropores of supports during the coating process, an effective approach is infiltrating the supports by high-volatile solvents which are immiscible with the solvents of dope solutions before coating [67]. Taking an organophilic polymer dissolved in an organic solvent as an example, the supports can be first immersed in water to thoroughly intrude into their pores. The as-fabricated composite membranes are then obtained after evaporation of coating solvents followed by the removal of the solvents in the supports [72].

The improvement in separation performance and/or robustness of membranes can be achieved through physicochemical modifications. A most common and facile technique is the incorporation of either inorganic or hybrid nanofillers into polymeric matrices as mixed matrix membranes including physical blending and chemical grafting method. Technically, physical blending is the most frequently used method in which fillers can be simply and directly introduced into polymer matrices with vigorous stirring and/or sonication to suppress their agglomeration and to facilitate their uniform distribution [73, 74].

The dispersion of nanofillers into polymeric matrices is generally carried out by three different procedures: (i) nanofillers are dispersed into a solvent first, and subsequently polymer is added into the suspension [75, 76]; (ii) polymer is dissolved in a solvent, afterward, nanofillers are added into the polymeric solution [77, 78]; (iii) nanofillers are dispersed into a solvent and polymer is dissolved in the same solvent separately, and then the filler suspension and the polymeric solution are mixed [73, 79]. It is important to highlight that mechanical stirring and/or ultrasonication using an ultrasonic bath or a probe-type ultrasonic homogenizer is performed for a specific period of time to prevent the fillers from agglomeration or sedimentation [14]. Besides, the remaining fabricated steps are essentially the same as those of aforementioned pure polymer membranes.

In some cases, to ameliorate the interfacial compatibility of two phases and/or prevent fillers from agglomerating, the fillers should be modified prior to addition. For instance, coupling agents are utilized as intermediates to form durable covalent bonds between inorganic fillers and polymer matrices [80]. It is worth mentioning that apart from the loading amount, the physical and chemical properties of fillers, such as size, shape, and functional groups on the surface, are deemed to dramatically impact membrane performance [19, 81–91]. In practice, a good number of nanofillers have been employed in conjunction with polymers to enhance their PV performance for ethanol recovery including zeolites (e.g., silicalite-1 and ZSM-5) [76, 92], fumed silica [93], metal–organic frameworks (MOFs) [94], zeolitic imidazolate frameworks (ZIFs) [95–97], carbon nanotubes [98], carbon blacks [99, 100], graphene-like fillers (graphene and graphene oxide derivatives) [101, 102], and polyhedral oligomeric silsesquioxanes (POSSs) [103].

Compared to flat-sheet membranes, hollow-fiber membranes used in PV separation offer some attractive advantages, i.e., higher space efficiency and self-supporting structure. A hollow-fiber membrane is shaped like a soda straw with a thin dense selective layer on the outside surface along with a microporous structure in the lumen side. Hollow-fiber membranes are commonly fabricated via an extrusion process, referred to as “spinning” including solution spinning and melt spinning [104]. Until now, few hollow-fiber membranes are, until recently, available for ethanol recovery application by PV. At present, the gain of an ideal multi-phase membrane-forming system and the exploitation of a corresponding well-controlled membrane fabrication procedure to achieve a high-performing hollow-fiber membrane have become a rising research area in hollow-fiber spinning [105].

## Membrane materials for ethanol recovery

Theoretically, the ethanol recovery from water by PV involves three effects: concentration polarization, coupling between mixed components as well as membrane swelling [106]. Concentration polarization, as an inherent phenomenon in the membrane separation process, denotes the formation of concentration gradients in the vicinity of membrane surface and bulk feed liquid due to the enrichment of rejected species (water) at the upstream membrane surface but also the decline of permeating species (ethanol). If ethanol molecules in the bulk feed are to permeate through the membrane, they have to diffuse through the concentration polarization region, referred to as the boundary layer, to the membrane surface. It definitely impacts the permeation of ethanol in a negative way. Higher feed flow velocity can effectively promote turbulence for a better mixing, bring ethanol molecules to the surface, and subsequently prevent water from further excessive accumulation, resulting in reducing the thickness of the diffusion boundary layer, thereby restraining this effect as much as possible [107]. The coupling effect means that the diffusion rates of ethanol and water influence each other during the PV progress. Ethanol and water molecules are bound together by hydrogen bonds. These combined molecules cause ethanol permeation resistance in the membrane, and are partially restricted and not allowed to leave the membrane. An increase in the affinity of the membrane towards ethanol over water would foster ethanol permeation, thus mitigating the coupling effect [108]. The swelling effect refers to the increase in the volume of membranes resulted from the absorption and accumulation of ethanol. Especially, many of polymeric membranes suffer from excessive swelling which breaks the regular arrangement of molecular chains, inducing a terrible change in the membrane structure. As a result, the membrane selectivity and the service life are deteriorated. Some strategies such as crosslinking and addition of fillers can prevent the excessive build-up of ethanol so as to suppress the ethanol-induced swelling behavior [109].

Given these discussions provided herein, it is obvious that the approaches for circumventing the coupling and swelling effect are all related to membranes. Hence, membrane materials play crucial roles not only in permeability and selectivity but also in overall properties including swelling resistance and mechanical strength. Till now, a wide variety of membrane materials have been exploited for the removal of ethanol from aqueous solutions by PV. Generally, ethanol perm-selective PV membranes can be classified into polymeric membranes, inorganic membranes, and mixed matrix membranes.

### Polymeric membranes

Hydrophobic polymers are identified as the most versatile and prospective membrane materials in PV application for ethanol extraction from water on account of their better malleability and processability as well as lower production costs. In addition, they have attracted widespread research interest. To date, many ethanol-selective polymeric membranes have been reported, including polydimethylsiloxane (PDMS), poly(1-trimethylsilyl-1-propyne) (PTMSP) and so on.

#### PDMS membranes

PDMS, usually referred to as ‘‘silicone rubber’’, is the most representative, well-studied, and widely used membrane material [110]. PDMS delivers an ethanol selectivity as a result of its backbone comprised entirely of Si–O bonds. It is considered as the benchmark material to recover ethanol from aqueous solutions. In addition, for now, from the aspect of the development of membranes selectively recovered ethanol in recent decades, it appears that PDMS would not be edged out of the top spot in the short term. In addition, PDMS also possesses a good membrane-forming property which is more prone to fabricate more practical configurations, such as common flat sheet and hollow fiber. A comprehensive review reported by O’Brien group on the economic analysis of ethanol production by a PV process coupled with a fermentor has concluded that a membrane with a permeation flux of over 150 g·m^−2^·h^−1^ as well as a separation factor of no less than 10.3 would be cost-competitive with distillation alone on a commercial scale [111]. In fact, PDMS-based membrane products in the ethanol removal application have been so far marketed, e.g., Pervap, Pervatech, PolyAn, and SolSep manufactured by Sulzer Chemtech, Pervatech BV, PolyAn GmbH, and SolSep BV, respectively. Unfortunately, Kujawski et al. found that the membrane Pervap 4060 presented the separation efficiency with the ethanol flux of 276 g·m^−2^·h^−1^, the water flux of 969 g·m^−2^·h^−1^, and the lower separation factor of 6 in a 4 wt. % of ethanol in water at 60 °C [64]. Similar lower separation factors were obtained for other commercial membranes under different test conditions [56, 65, 66], as summarized in Table 1.

**Table 1:** Ethanol–water separation performance of PDMS membranes

Membrane material/support	Feed concentration (wt. %)	Feed temperature (°C)	Separation factor	Flux (g·m^−2^·h^−1^)	References
Pervap 4060	5	50	7.1	1300	[55]
Pervap 4060	5	25	7	138	[56]
Pervap 4060	4	25	4	2078	[64]
Pervatech	5	50	6.7	2600	[55]
Pervatech	5	25	6	184	[56]
Pervatech	3	30	5	1608	[65]
PolyAn	5	25	6	369	[56]
SolSep 3360	8.7	33	7	1140	[66]
PDMS	1.5	66	10.4	150	[85]
PDMS	5	90	9	1906	[124]
PDMS	6	50	8.6	100	[125]
PDMS/PSF	10	60	11.6	1493	[48]
PDMS/PSF	5	60	8.2	1186	[93]
PDMS/PSF	4	45	5	1600	[113]
PDMS/ceramic	5	40	8.9	1600	[70]
PDMS/tubular zirconia/alumina ceramic	4.2	60	7.9	4190	[23]
PDMS/PES	5	40	5.5	1124	[126]
PDMS/PA	4	45	~ 8.5	~ 1850	[113]
PDMS/PA	10	40	5	160	[127]
PDMS/CA	3	50	1	2800	[45]
PDMS/CA	5	40	9.3	1140	[46]
PDMS/CA	5	40	8.5	1300	[22]
PDMS/CA	3	30	3.7	1060	[128]
Self-assembled monolayer-modified PDMS/PSF	5	60	13.1	413	[69]
Multiple sprayed PDMS/PSF	5	60	7.5	3275	[129]
PDMS/PI	3	41	4.6	120	[74]
PDMS/PVDF	9	60	11.2	1329	[43]
PDMS/PVDF	5	50	8.5	265	[44]
PDMS (prepared in water phase)/PVDF	3	30	11.5	449	[121]
Copoly(IPAA-FA)–PDMS blend	2.5	24	19.7	160	[114]
PDMS–HMDSO blend/PDMS-*g*-PVDF	10	35	5.1	1300	[115]
PDMS-*b*-PPO copolymer	5	60	8.5	3817	[116]
PDMS-*b*-PSSQ copolymer	5	30	11	106	[117]
SDS triblock copolymer	5	76	8.8	540	[118]
PDMS-PS-poly(4-hydroxystyrene) block copolymer/PVDF	8	25	6.8	4500	[130]
PDMS-*g*-phenylpropyne copolymer	7.3	30	22.5	553	[131]
PDMS-*g*-PS copolymer (dip-coating both sides of the PTFE support)	8.1	60	~ 8.5	~ 300	[49]
Segmented polyurethaneurea containing PDMS	10	40	8.6	130	[49]
PDMS-*g*-copolyimide PIS19	9.53	50	9.5	5720	[119]
Cross-linked PDMS	9.60	50	9.8	2690	[119]
PDMS plasma treated with octadecyldiethoxymethylsilane	4	25	16.3	16	[120]
F-PBZ-modified PDMS	5	50	5.1	215	[132]
PDMS impregnated into pores of PTFE	1.5	66	8.3	760	[85]
SBS/PDMS/F-42	3	50	7.5	162	[68]
Copoly(PDMS-phosphate ester)/TMVS-*g*-PVDF	10	–-	4.6	2850	[122]
Copoly(PDMS-phosphate ester)/TMVS-*g*-PVDF (multi-layer)	10	–-	31	900	[123]
PDMS (commercial hollow fiber)	3	30	10.6	6	[62]
PDMS/PSF (hollow fiber)	8	50	6.4	265	[63]
PDMS/PEI (hollow fiber)	4.6	30	8.4	243	[50]

*PSF* polysulfone, *PES* polyethersulfone, *PA* polyamide, *CA* cellulose acetate, *PI* polyimide, *PVDF* polyvinylidene fluoride, *IPAA-FA* copoly(N-isopropylacrylamide/1H,1H,2H,2H-perfluorododecyl acrylate), *HMDSO* hexamethyldisiloxane, *PPO* polyphenylene oxide, *PSSQ* phenylsilsesquioxane, *SDS* polystyrene-*b*-PDMS-*b*-polystyrene, *PS* polystyrene, *PTFE* polytetrafluoroethylene, *F-PBZ* fluorinated polybenzoxazine, *SBS* styrene–butadiene block copolymer, *TMVS* trimethoxyvinylsilane, *PEI* polyetherimide.

Besides, Beaumelle reviewed that pristine PDMS membranes generally delivered lower ethanol/water separation factors of less than 10 along with a broad range of permeation fluxes ranging from 1 to 1000 g·m^−2^·h^−1^ [112]. Similarly, O’Brien also indicated that ethanol/water separation factors for ‘pure’ PDMS membranes were in a range of 4.4–10.8. This significant variation in values is mainly related to the molecular weight, crosslinking density, membrane thickness, potential support layer, operating condition, etc. From these analyses, it is clear that the ethanol recovery performance of pure PDMS are expected to be elevated.

To this end, many efforts have been spent on the modification of PDMS membranes. A simpler way is to prepare composite membranes by coating thin PDMS active layers on highly porous supports. Through reducing the effective thickness, the permeation flux can be improved, as listed in Table 1. For example, permeation fluxes of composite membranes supported on polysulfone (PSF) [113], polyamide (PA) [113], and cellulose acetate (CA) [45] were achieved as high as 1600, 1850, and 2800 g·m^−2^·h^−1^, respectively.

In addition, PDMS modification by blending, blocking, or grafting with other polymers has been attracting considerable attention. A blend membrane consisting of PDMS and 5.3 wt. % copoly(N-isopropylacrylamide/1H,1H,2H,2H-perfluorododecyl acrylate) (IPAA-FA) showed a higher separation factor (19.7) for a 2.5 wt. % ethanol/water mixture [114]. Chang et al. blended PDMS with hexamethyldisiloxane (HMDSO) cured by phenyl triethoxysilane, then coated the silicone layer on the microporous PVDF support on which PDMS was plasma-induced grafted for enhancing the adhesion. Although the as-prepared loose network structural composite membrane displayed a lower separation factor of 5.1, the corresponding permeation flux was up to approximately 1300 g·m^−2^·h^−1^ [115].

For block copolymerization, Liu reported a PDMS-*b*-polyphenylene oxide (PPO) block copolymer membrane. Its permeation flux could reach a high of 3817 g·m^−2^·h^−1^, along with a separation factor of 8.5 when concentrating a 5 wt. % ethanol aqueous solution [116]. Guo et al. prepared membranes with a novel silicone block copolymer which was synthesized by the condensation of rubbery PDMS with glassy ladder like phenylsilsesquioxane (PSSQ). The unique cage-type structure of the PSSQ mitigated the swelling of the membrane and enhanced the affinity of the membrane towards ethanol. The PV results showed that the maximum separation factor afforded by the prepared membranes was 11 at a 5 wt. % ethanol solution [117]. Indeed, it was improved as compared with that of pristine PDMS membranes. In addition, a microphase-separated triblock copolymer–polystyrene (PS) -*b*-PDMS-*b*-PS (SDS) was reported to prepare membranes for recovery of volatile organic compounds (VOCs) from aqueous mixtures by PV. It was proven that both the permeation flux and separation factor offered by these SDS membranes were closely dependent on ethanol concentration in feed mixtures. When separating a 5 wt. % ethanol binary aqueous solution, a permeation flux of 540 g·m^−2^·h^−1^ and a separation factor of 8.8 were observed [118].

With regard to graft copolymerization, Nagase et al. successfully synthesized three kinds of PDMS-graft copolyimides with different PDMS segment lengths (labeled as PIS6, PIS11, and PIS19, respectively) by polycondensation, and used them to make the tough self-standing membranes. It was observed that all of these membranes exhibited perm-selectivity toward ethanol as well as the other organics along with stable and incredibly high permeability. In particular, the PIS19 offered the best PV performance with the moderate separation factor of around 9.5 and the very advantageous permeation flux of 5720 g·m^−2^·h^−1^ which was at least more than sixfold that of PDMS alone [119].

Similar to graft copolymerization, surface modification with a silane compound is also an effective approach to maximize the membrane ethanol-selectivity. Kashiwagi and co-workers treated PDMS membranes utilizing plasma grafting with the silane compound octadecyldiethoxymethylsilane containing a long alkyl chain. The treated membrane yielded a maximum separation factor of 16.3 at 25 °C [120].

Other PDMS-based membranes reported in the literature have primarily been concentrating on incorporating fillers to fabricate mixed matrix membranes. The related studies will be discussed later in “Mixed matrix membranes”. Meanwhile, significant efforts have been positioned toward exploiting novel fabrication methods. A PDMS membrane was fabricated by Qin’s group in the water phase instead of the conventional organic phase with dodecylbenzenesulfonic acid (DBSA) as surfactant and PVDF as support for the recovery of ethanol from model ethanol aqueous solution and fermentation–PV integrated process, respectively. The permeation fluxes fell in the range of 396–664 g·m^−2^·h^−1^ and 332–548 g·m^−2^·h^−1^, and the separation factors were in the range of 8.6–11.7 and 8–11.6 in the fed-batch and continuous fermentation–PV system, respectively [121]. Wang et al. unveiled a facile and scale-up roll-coating method by which multi-layer PDMS was easily and controllably assembled on top of the PSF support. The as-fabricated pilot-scale composite membranes were adopted to separate ethanol by PV in a lab scale and a pilot plant. The PV results showed a high and stable PV selectivity towards ethanol over water with a separation factor of 11.6 as well as a permeation flux of 1493 g·m^−2^·h^−1^, which was excellent as compared with the corresponding values for the PDMS/PSF membranes prepare by other methods. It was also concluded that this method was instructive in obtaining defect-free membranes and scaling up to larger systems [48]. A membrane reported by Mori et al., impregnated PDMS into the pores of polytetrafluoroethylene (PTFE) support, can be thinned to 5 μm in thickness. In this case, it gave the permeation flux and separation factor corresponding to 760 g·m^−2^·h^−1^ and 8.3, respectively [85].

Moreover, constructing an architectural multilayer structure is also an ingenious way. More recently, Figoli and co-workers explored a three-layer asymmetric flat-sheet composite membrane, in sandwich-like configuration, which consists of a dense top layer of styrene–butadiene block copolymer (SBS) and an intermediate layer of PDMS or polyurethane (PU) layer followed by a support layer of fluoroplast F-42 with high porosity via layer-by-layer assembly. An increase in ethanol selectivity of the composite membranes with PDMS as the intermediate layer was observed with a maximum of 8.3 in an aqueous solution containing 3 wt. % ethanol at 30 °C. On the other hand, the membranes also showed a strong increase in permeation flux as a result of the lower membrane thickness of selective layers [68]. Chang and Chang synthesized a copolymer of PDMS and phosphate ester as the selective membrane material [122], and designed a multi-layer membrane configuration by employing alternating layers of dense copoly(PDMS-phosphate ester) silicone and porous PVDF on which trimethoxyvinylsilane (TMVS) was previously plasma-polymerized to form a thin and loose polymeric layer. It was confirmed that the multiple-layer composite membranes exhibited excellent PV performance, especially at lower ethanol concentration. Within a specific reference, the more the silicone layers were, the higher the separation factor, but the lower the permeation flux was due to the trade-off effect. In particular, the four-layer PDMS membrane delivered the optimal separation capability with an exceptional separation factor of 31 and a high permeation flux of 900 g·m^−2^·h^−1^ in a 10 wt. % ethanol-containing aqueous solution [123].

Several researchers have reported the PDMS membranes in hollow-fiber configuration. The commercial PDMS hollow-fiber membranes as well as the PVC membrane module manufactured by PAM-Membranas Selectivas in Brazilian were chosen by Marangoni et al. to selectively remove ethanol from actual fermentation broth with 3 wt. % of ethanol. Notably, the permeation flux was only about 6 g·m^−2^·h^−1^ [62]. Apparently, this value was incredibly lower although it was essentially in accordance with the datum provided by the supplier. In contrast, the PDMS hollow-fiber membranes with supporting materials, such as PSF and polyetherimide (PEI) as gathered in Table 1, achieved relatively higher permeation fluxes. Nevertheless, these data are still lower than those of PDMS-base flat-sheet membranes. The complexity of the hollow-fiber fabrication process and the limitation of the optimal combination of strength and performance result in limited offerings.

#### PTMSP membranes

Another widely studied polymeric membrane material for this application is poly [1- (trimethylsilyl) -1-propyne] (PTMSP). It is a super glassy polymer with better processability and scalability than rubbery ones like PDMS. In addition, it possesses an extra high free volume which is associated with a higher flux of permeates. The data of ethanol/water PV performance reported in the literature references for pristine PTMSP membranes are tabulated in Table 2. It is observed that the ethanol separation factor, shown in the fourth column, is ranged from 9 to 26, which is much larger than that of pure PDMS. Unfortunately, a large number of experimental studies have proved that such super glassy polymers suffer from rapid physical aging which is a ubiquitous phenomenon since polymer chains undergo physical relaxation with time and are prone to converge towards a thermodynamic equilibrium [133], resulting in the reduction in free volume [134]. Under these circumstances, the separation performance of PTMSP membranes in terms of both permeability and selectivity decay continuously over time. To complicate matters, this decline becomes more serious for thinner membranes, which restricts the increment in permeation flux. Gonzalez-Velasco et al. calculated the decreasing tendency of ethanol selectivity with time based on the experimental data. They demonstrated that, for a PTMSP membrane of 100-μm thickness, the separation factor fell to a level of approximately 8 from the initial value of around 10.7 after continuous exposure to a 10 wt. % ethanol aqueous solution at 75 °C for 450 h. Though the separation factor maintained practically constant over a 40-h period, the permeation flux dropped from 540 to 350 g·m^−2^·h^−1^ [135]. A similar result that the selectivity for a 30-μm PTMSP membrane could only remain at about the initial level for just 40 h was attained by Masuda [136]. This severe long-term instability is further aggravated for concentrated solutions and higher temperatures due to the higher mobility of polymer chains. Gonzalez-Velasco assessed the deterioration of PTMSP over a long operation time of 572 h in a higher feed concentration of 50 wt. % at higher temperatures of 50 and 75 °C. Experimentally, it was observed that both the separation factor and permeation flux of PTMSP membranes in various thickness diminished rapidly in a short time, then reduced slowly for test time longer than 250 h. Eventually, when operating at 50 °C, the separation factor and permeation flux for the 52-μm membrane decreased to 3 and 800 g·m^−2^·h^−1^ from the initial value of around 4.5 and 1200 g·m^−2^·h^−1^, respectively [137]. It is because of this unfavorable characteristic, the commercial application of PTMSP remains limited at this time.

**Table 2:** Ethanol–water separation performance of PTMSP membranes

Membrane material	Feed concentration (wt. %)	Feed temperature (°C)	Separation factor	Flux (g·m^−2^·h^−1^)	References
PTMSP	6	60	10–26	224–1150	[57]
PTMSP	5	50	14–24	300–7600	[55]
PTMSP	6	25	22.9	52	[150]
PTMSP	6	30	19.9	330	[146]
PTMSP	1.5	66	18.7	1570	[85]
PTMSP	10	50	~ 17	~ 800	[147]
PTMSP	10	50	14.5	210	[151]
PTMSP	10	75	10.7	540	[135]
PTMSP	6.6	50	10.3	480	[139]
PTMSP	5	30	9	389	[152]
PTMSP	10	40	8.3	188	[153]
PTMSP-*g*-PDMS copolymer	7	30	28.3	61	[138]
Trimethylsilylated PTMSP	6.2	50	17.6	590	[139]
*n*-Decyldimethylsilylated PTMSP	6.1	50	17.8	430	[139]
PTMSP/PFA-*g*-PDMS	10	40	20	600	[141]
PTMSP-PDMS (semi-IPN method)	5	30	13.7	600	[142]
PTMSP-PDMS (PDMS sorption method)	5	30	12	250	[142]
PTMSP-PDMS (PDMS sorption and crosslinking method)	5	30	8	150	[142]

*PFA* poly(fluoroacrylate), *IPN* interpenetrating polymer network

In this regard, several methods have been put forth to stabilize the PTMSP membranes and to further improve the ethanol-selective separation performance for brightening the application prospects of this material. At the very beginning, Nagase’s group successively attempted to optimize PTMSP performance by integrating short PDMS chains [138], trialkylsilyl groups [139], and fluoroalkyl groups [140]. In particular, all PTMSP membranes grafted with PDMS in any proportion delivered higher selectivity to ethanol than PTMSP and even the most outstanding PDMS alone. The separation factor reached a maximum of 28.3 for the copolymer membrane containing 12 mol % of PDMS, while the corresponding permeation flux was mere 61 g·m^−2^·h^−1^ which was not ideal or even discouraged [138]. From these researches, it is inferred that polymer design by block or graft copolymerization would probably be an effective and flexible way. Furthermore, the blend method is also a promising strategy, including polymer blend and integration inorganic or hybrid nanofillers reviewed in “Mixed matrix membranes”. Uragami and co-workers added the poly(fluoroacrylate) (PFA)-*g*-PDMS graft copolymer into the dope solution to enhance the surface hydrophobicity of PTMSP membranes. The PDMS chains of PFA-*g*-PDMS were trapped in the PTMSP matrix, while the PFA at the other end of PFA-*g*-PDMS was mainly localized on the membrane surface just like the grass on the ground. The surface-modified PTMSP membranes displayed higher water repellency, leading to lower solubility of water, therefore, higher ethanol permselectivity [141].

Subsequently, Kang et al. also employed PDMS to circumvent the intrinsic issue without sacrificing permeability if possible. Three preparation methods, semi-IPN, PDMS sorption, and PDMS sorption and crosslinking, were carried out. Although the initial permeation flux could be up to 600 g·m^−2^·h^−1^ along with a modest separation factor of around 13.7 by semi-IPN method, it quickly reduced to 200 g·m^−2^·h^−1^ after a 7-day run along with a strikingly reduced separation factor of 7.3. In contrast, the membranes prepared by PDMS sorption as well as PDMS sorption and crosslinking presented more stable permeable behavior with time, but their separation properties were lower [142]. As reviewed herein, it appears that future research focusing on developing novel preparation methods of PTMSP-based membranes is a reasonable approach to retard the physical aging.

Since Masuda et al. systematically reported the impact of polymerization conditions on properties of PTMSP in the 1980s [143–145], almost all the PTMSP has been synthesized according to their technique, using metal catalytic systems. As confirmed by Volkov, overall properties of PTMSP were strongly correlated with catalyst. Despite a combination of high permeation flux (over 300 g·m^−2^·h^−1^) and high separation factor (over 15) for all PTMSP membranes synthesized with three catalytic systems (TaCl_5_/*n*-BuLi, TaCl_5_/Al(*i*-Bu)_3_, and NbCl_5_), the permeation flux and separation factor for TaCl_5_/*n*-BuLi-catalyzed PTMSP membranes deteriorated significantly with time; conversely, those for the membranes synthesized by the other two catalysts, TaCl_5_/Al(*i*-Bu)_3_ and NbCl_5_, could remain stable in either synthetic acetic acid-containing aqueous solutions or actual fermentation broths [146]. More than 3 decades later, the most common catalysts for PTMSP synthesis nowadays are still TaCl_5_ [135, 147], NbCl_5_ [148], and TaCl_5_/Al(*i*-Bu)_3_ [58, 149]. Hence, the exploitation of active catalysts should deserve more attention.

#### Other polymeric membranes

Apart from widely known PDMS and PTMSP, a considerable amount of effort has been placed in searching for polymeric materials with higher ethanol perm-selectivity, higher permeation fluxes, and better physical properties. Unfortunately, a few membrane materials are available, and the separation performance of most of them is not as good as expected. A list of other polymeric materials and respective separation data from the literature for ethanol removal from water is assembled in Table 3.

**Table 3:** Ethanol–water separation performance of other polymeric membranes

Other polymeric membrane material	Feed concentration (wt. %)	Feed temperature (°C)	Separation factor	Flux (g·m^−2^·h^−1^)	References
Plasma-polymerized silicone	4	25	1.5–5.2	180–380	[120]
Plasma-polymerized silane compounds	4	25	13.2–16.9	5.3–21	[120]
Polyhydromethylsiloxane oil	4	25	14.4	26	[120]
Plasma-polymerized hexamethyltrisiloxane plasma treated with octadecyldiethoxymethylsilane	4	25	18	15	[120]
PMES	4.4	50	10.5	114	[154]
PMPS	4.1	50	11.7	134	[154]
PPMS	5	40	6.2	1433	[46]
PSI (synthesized from ODMS, PMDA, and MDMS)	10	40	10.6	560	[156]
PSI (synthesized from ODMS, 6FDA, and MDMS)	10	40	3.6	2120	[156]
POMS	4.7	53	8.3	670	[157]
POMS	5	50	3.95	120	[158]
POMS	5	30	5.7	235	[159]
PVTES	9	45	3.8	14,059	[160]
PVTES-DMDES	9	35	5.6	6909.3	[161]
PVTES-HSO	9	35	6.6	8160.1	[161]
PVTES-PDMS	9	35	6.3	539.8	[161]
Structured siloxane-containing copolymer	30	16	7.4	70	[162]
Styrene-*g*-fluoroalkyl acrylate copolymer/PDMS	8	30	45.9	5	[163]
PFP	4.8	40	7	300	[165]
PEBA 2533	5	23	2.5	118	[166]
Polyphosphazene with substituted –OCH_2_CF_3_ group	10	40	6.1	260	[127]
Poly(acrylonitrile-*co*-methyl acrylate)	25	-	11.03	1.3	[167]
PIM-1	10	60	9.3	1400	[168]
PIM-1	5	65	3.1	700	[20]
Porous PTFE	2	60	2.25	10,592	[170]
PA	20	22	1.6	248	[175]
PVDF (hollow fiber)	5	50	5–7.8	3500–8800	[172]
Trioctylamine liquid membrane immobilized in porous PP hollow fiber	5	54	32	5.1	[173]
Trioctylamine liquid membrane immobilized in porous PP hollow fiber	10 added 2.5 wt. % n-butanol	54	113	17	[51]
SBS (dense)	3	41	5.5	146	[74]

*PMES* polymethylethoxysiloxane, *PMPS* polymethylphenylsiloxane, *PPMS* polyphenylmethylsiloxane), *PSI* polysiloxaneimides, *ODMS* α,ω-(bisaminopropyl) dimethylsiloxane, PMDA 1,2,4,5-benzenetetracarboxylic dianhydride, *MDMS* 1,3-bis(3-aminopropyl)tetramethyldisiloxane, *6FDA* 5,5-[2,2,2-trifluoro-1-(trifluoromethyl) ethylidene] bis-1,3-isobenzenefurandione, *POMS* polyoctylmethyl siloxane, *PVTES* poly(vinyltriethoxysilane), *DMDES* dimethyldiethoxysilane, *HSO* hydroxy silicone oil, *PFP* perfluoropropane, *PEBA* poly(ether block amide), *PIM* polymers of intrinsic microporosity, *PTFE* polytetrafluoroethylene, *PA* polyamide, *PVDF* polyvinylidene fluoride, *PP* polypropylene, *SBS* styrene–butadiene block copolymer.

In summary, the reported ethanol perm-selective materials are mostly based on homopolymers or copolymers of siloxanes with hydrophobic Si–O–Si backbone and the separation performance is strongly correlated to siloxane which provides excellent separation potential. At the very beginning, Kashiwagi et al. synthesized a series of ethanol-permeable membranes by plasma polymerization with various silicone and silane monomers. These membranes had PDMS-like structures and their separation factors and permeation fluxes were in the range of 1.5–5.2 and 180–380 g·m^−2^·h^−1^, respectively. Meanwhile, they utilized silane compounds with long-chain alkyl groups to prepare silane membranes. Despite showing gratifying separation factors ranging from 13.2 to 16.9, permeation fluxes afforded by the polymerized silane membranes were relatively low in the 5.3–21 g·m^−2^·h^−1^ range. Similarly, their plasma-polymerized silicone oil membranes or the ones further treated with octadecyldiethoxymethylsilane all showed discouraging separation results [120].

Thereafter, two room-temperature vulcanizing-type (RTV) silicone rubbers, polymethylethoxysiloxane (PMES) and polymethylphenylsiloxane (PMPS), were employed by Chen et al. for the fabrication of ethanol-selective membranes. Simultaneous increments in both permeation flux and separation factor for them were achieved by comparison with PDMS. Especially, the separation factors multiplied even more than two times (up to 10.5 and 11.7 from 5.3) when operating at 50 °C [154, 155]. Meanwhile, Li et al. prepared polyphenylmethylsiloxane (PPMS) membranes with the same backbone as PDMS. The resultant membranes yielded a higher permeation flux, but a lower separation factor as compared with PDMS prepared in the same way attributing to the presence of the more hydrophobic and rigid phenyl groups in PPMS [46].

Krea et al. synthesized polysiloxaneimides (PSI) block copolymers with high contents of siloxane block in the 70–95 wt. % range. They found that the higher the siloxane content was, the better the PV performance. At the 94 wt. % PDMS content and 1.5:2:0.5 equivalents of aminopropyl siloxane (ODMS):1,2,4,5-benzenetetracarboxylic dianhydride (PMDA):1,3-bis(3-aminopropyl) tetramethyldisolxane (MDMS), the as-fabricated PSI copolymer membranes displayed the optimum PV performance (separation factor of 10.6 and permeation flux of 560 g·m^−2^·h^−1^) when carrying out in a 10 wt. % ethanol solution at 40 °C [156].

Besides, the GKKS Research Center in Germany developed a hydrophobic polyoctylmethyl siloxane (POMS) membrane on a polyacrylonitrile (PAN) support. Several groups employed their POMS membranes to separate binary ethanol aqueous solutions. Garcia et al. observed that the POMS membrane displayed a permeation flux of approximately 670 g·m^−2^·h^−1^ accompanied with a moderate separation factor of about 8.3 at 53 °C with a feed containing 4.7 wt. % ethanol in water. In addition, the authors indicated that the membrane permeability was related to the preferential sorption, whereas the selectivity was related to the solubility rather than diffusivity [157]. However, the result carried out by Lazarova et al. showed that the permeation flux and separation factor for the POMS membranes were 120 g·m^−2^·h^−1^ and 3.95, respectively, when exposing in a 5 wt. % ethanol feed at 50 °C [158]. In contrast, Straathof and co-works obtained intermediate data (the permeation flux of 235 g·m^−2^·h^−1^ and the separation factor of 5.7) in the same concentration of ethanol aqueous solution [159].

More recently, Zhang et al. prepared novel thin poly(vinyltriethoxysilane) (PVTES) membranes to recover ethanol. They unveiled that the resulting membranes delivered consumedly high permeation fluxes ranging from 6000 to 10,000 g·m^−2^·h^−1^, which clearly transcended the upper limit for PDMS membranes as shown in Table 1, while maintaining more constant separation factors of around 5 over feed concentrations range from 3 to 13 wt. % at 35 °C [160]. Subsequently, they continued their work on modifying the rigid structure of the PVTES membrane by copolymerization with dimethyldiethoxysilane (DMDES), oligomer of hydroxy silicone oil (HSO), and PDMS to optimize its separation performance. It was proved that the separation performance had the direct proportion with the chain flexibility and the amount of hydrophobic groups. Hence, the PVTES-HSO membrane showed the best PV performance which was the exceptionally high permeation flux of 8160 g·m^−2^·h^−1^ and moderate separation factor of 6.6 in a 9 wt. % ethanol aqueous solution at 35 °C. As summarized in Table 3, it appeared that PVTES, PVTES-DMDES, and PVTES-HSO membranes showed great potential for in situ recovery of ethanol [161].

It should be noted that the siloxane-based membranes prepared by conventional methods were all dense, where only intermolecular spacing between the chains, referred to as accessible free volume, provided the pathway for permeate transport. A synthetic membrane composed of the monomers of octamethyl cyclotetrasiloxane, vinyl heptamethyl cyclotetrasiloxane, styrene, and divinyl benzene was constructed by Shi et al. via the concentrated emulsion polymerization method, in which the gaps among the latex region and inside the dense latexes provided diffusional pathways for permeates. However, the structured membrane was much thicker than traditional dense ones, and its thickness was 750 μm, more than ten times as thick as the one with a uniform structure, and even more than that with a porous support. Although the separation performance of the resultant membranes was not as good as expected, displaying a permeation flux of 70 g·m^−2^·h^−1^ and a separation factor of 7.4 with a feed mixture of 30 wt. % ethanol at 16 °C, it has provided a new opportunity for the development of new material membranes [162].

Other polymeric membrane materials have been investigated by several research groups. For instance, Ishihara et al. successfully fabricated a composite membrane composed of a 20-μm styrene (St)-fluoroalkyl acrylate (FAA) graft copolymer skin layer and a 100-μm PDMS layer. The membrane presented the separation factor value as high as 45.9. They suggested that this was attributable to the relatively low affinity between the hydrophobic St-FAA graft copolymer and ethanol molecules, effectively suppressing the membrane swelling and resisting the dissolution of water. Due to the trade-off relationship between permeation flux and separation factor, the permeation flux of the St-FAA/PDMS composite membrane was only 5 g·m^−2^·h^−1^ [163, 164]. Later, Masuoka and co-workers fabricated plasma-polymerized perfluoropropane (PFP) membranes on porous PSF supports, and observed a permeation flux of 300 g·m^−2^·h^−1^ with a separation factor of 7 [165].

Liu et al. employed poly(ether block amide) (PEBA 2533) membranes for pervaporative recovery of ethanol from water. Depressingly, their observation showed that the separation performance was poor, especially the separation factor was as low as 2.5, owing to the weak affinity between ethanol and PEBA. What is worse, the permeation flux reduced to 37 from 118 g·m^−2^·h^−1^, more than three times, when thickening up to 100 μm in thickness from 30 μm [166].

A series of polyphosphazene heteropolymers with different hydrophobic pendant groups were produced by Huang et al. to fabricate membranes for ethanol/water separation. They found that the polyphosphazene membrane with –OCH_2_CF_3_ substituting groups showed the highest separation performance both in permeation flux and separation factor, which were, respectively, 260 g·m^−2^·h^−1^ and 6.1, owing to its highest affinity to ethanol, highest diffusivity, as well as highest diffusion selectivity in comparison with the other two membranes with –OC_2_H_5_ and –OCH_2_CF_2_CF_2_CF_2_CF_2_H groups [127]. It was concluded that enough affinity of ethanol toward the membrane materials and enough high diffusivity across the membranes were indispensable for the ideal membranes.

Abu-Saied’s group fabricated poly(acrylonitrile-*co*-methyl acrylate) membranes for ethanol extraction from aqueous mixtures and bioethanol purification from fermentation broth originating from cellulosic fiber wastes both in the laboratory scale [167]. It was found that the polymeric membrane was hydrophobic and ethanol-selective. Under a nitrogen pressure value of 40 psi, the separation factor of the resultant polymeric membrane was found to reach a maximum value of 11.03 with an appreciably poor permeation flux of 1.3 g·m^−2^·h^−1^ for a mixture of 25 wt. % ethanol. On the other hand, the membrane exhibited an optimal separation factor of 9.27 and a similarly low permeation flux of 1.4 g·m^−2^·h^−1^ with a 33% fermentation system.

More recently, glassy PIM-1, one of the so-called polymers of intrinsic microporosity (PIMs), was employed in this particular application in terms of its organophilicity and intrinsic microporosity. Adymkanov and co-workers reported that the PIM-1 membranes with thicknesses ranging from 25 to 40 µm exhibited a maximum selectivity of 10.7 and a permeation flux of 470 g·m^−2^·h^−1^ in a 10 wt. % dilute aqueous mixture at 30 °C. In addition, the permeation flux could be as high as 1400 g·m^−2^·h^−1^ corresponding to a marginally lower separation factor of 9.3 when the operating temperature rose to 60 °C [168]. However, a recent study by Alberto et al. showed that a 60 ± 9-µm-thick PIM-1 membrane possessed an appreciably lower separation factor (3.1 ± 1.7) and a relatively higher permeation flux (circa 700 g·m^−2^·h^−1^) than the pure PTMSP membrane [20]. It seemed that such membrane was not very selective for ethanol. Unfortunately, glassy polymers, such as PIM-1 and PTMSP, have been proven to suffer from physical aging that is the long-term operating instability. Furthermore, the polymeric PIM-1 membrane also has serious excessive swelling in the presence of ethanol [169]. These problems would severely stymie its application in ethanol extraction.

In addition to the aforementioned nonporous polymeric membranes, the porous membranes also have been proposed to recover ethanol via the PV process. Nakao et al. applied a commercially available microporous PTFE membrane (0.2 μm pore diameter) into a continuous ethanolic fermentation system. It was demonstrated that the PTFE membrane displayed an attractive permeation flux with a value of 5700 g·m^−2^·h^−1^, whilst the extracted ethanol concentration was 6–8 times higher than that in feed [12]. Aroujalian et al. also applied the microporous PTFE membrane with the same pore diameter to separate ethanol/water mixtures. The maximum values of 10,592 g·m^−2^·h^−1^ for permeation flux was achieved at 60 °C, but with a rather poor separation factor of 2.25 [170]. On the other hand, Sukitpaneenit and co-workers explored and reported an asymmetric PVDF hollow-fiber membrane produced by the dry-jet wet spinning for ethanol/water separation. It possessed a porous inner surface, a thicker finger-like macrovoid cross-section, and a rough outer surface with small voids or defects, thus yielding a superior permeation flux of roughly 8000 g·m^−2^·h^−1^ [171]. Moreover, the membrane performance was strongly restricted by morphology, pore size, as well as pore distribution which could be controlled by altering spinning conditions. Overall, the membrane with the long finger-like macrovoid structure displayed a satisfactory permeation flux (8795 g·m^−2^·h^−1^) with an associated separation factor of near 5; while the membrane with the nearly macrovoid-free morphology presented an improved separation factor of around 7.8 and an inevitably and considerably reduced permeation flux (3961 g·m^−2^·h^−1^) [172]. In general, porous membranes exhibited low selectivity, but high permeability due to their porosity.

In contrast, microporous hydrophobic membranes could be employed as supports to immobilize liquids with high boiling points so as to form liquid membranes to achieve high ethanol-selectivity. Thongsukmak and Sirkar immobilized trioctylamine into the pores of a polypropylene (PP) hollow-fiber membrane and coated a nanoporous fluorosilicone coating on the outside. Their study has demonstrated the liquid membrane had long-term operational stability over 300 h. For a binary mixture containing 5 wt. % ethanol, it had a high separation factor (maximum 32), but an extremely low permeation flux of 5.1 g·m^−2^·h^−1^ [173]. When adding 2.5 wt. % n-butanol into a feed of ~ 10 wt. % ethanol solution, the permeation flux slightly went up to 17 g·m^−2^·h^−1^. However, the separation factor increased conspicuously to as much as 113, namely, the concentration of organic compounds in the permeate exceeded 95%. In addition, they evaluated the performance of a thinner trioctylamine liquid membrane, and their results confirmed that the permeation flux and separation factor were approximately 65 g·m^−2^·h^−1^ and 100, respectively, for separation of a dilute aqueous ethanol (~ 10 wt. %) and butanol mixture (2 wt. %) [51]. Notably, the major problem restricting the widespread application of liquid membranes is stability because of various losses. Further, the lost liquid membrane compounds to the fermentation broth are most probably toxic to the yeast cells [174]. Accordingly, only perfectly ensure the stability of liquid membranes and hence prevent the fermentation broths from contamination can liquid membrane-based PV become a potential separation technology in separating ethanol from aqueous solutions.

As reviewed above, reported ethanol/water separation factors for polymeric materials other than PDMS and PTMSP cover a fairly broad range, from 1.5 to 113, with reversely changed permeation fluxes ranging from 5 to over 14,000 g·m^−2^·h^−1^ attributing to the intrinsic trade-off effect. Collectively, new film-forming materials with higher permeability and ethanol selectivity, as well as better chemical, thermal, and long-term operation stability, should be actively explored.

### Inorganic membranes

It is known that inorganic membranes, fabricated from ceramics or zeolites, are widely used today for dehydrating organic compounds via PV [176, 177]. Over the past 2 decades, however, some researchers have committed themselves to exploring inorganic membranes for recovery of ethanol from aqueous solutions because of their special and competitive superiority in separation performance (both separation factor and permeation flux), mechanical properties, chemical resistance, thermal stability, anti-fouling ability, and long-term durability over most polymeric ones.

Hydrophobic zeolites have attracted tremendous attention from the researchers for ethanol recovery application via PV. Zeolite consisting of hydrated aluminosilicate is an inorganic crystalline structure with a uniform pore diameter ranged in 0.3–1.3 nm. Zeolite membranes are most often fabricated by depositing a polycrystalline zeolite layer onto a porous inorganic support layer (like tubular or discoid alumina or stainless steel, and even ceramics) via hydrothermal synthesis method [178]. Generally, whether zeolite membranes are water-selective or organic-selective relies on their hydrophilicity/hydrophobicity. Hydrophilic zeolite membranes, such as A- and X-type zeolites, allow preferential permeation of water; whereas hydrophobic zeolite membranes, such as silicalite-1 and ZSM-5, preferentially permeate organics. During PV separation, permeating molecules first adsorb into the zeolite pores due to intermolecular attractive forces and then diffuse through the zeolite membrane, driven by the chemical potential gradient. It is obvious that their separation performance is closely related to the zeolite framework structure and pore size which can be adjusted by means of the change of the content of Al and other metals substituted into the framework (if any) as well as preparation conditions during the membrane fabrication process.

The MFI zeolite structure is most commonly deployed to prepare membranes to date, as compared with many other zeolite structures (more than 14, including MEL [179], MOR [180, 181], CHA [182, 183] and so on). This is not only due to its medium pore size (nearly 0.55 nm), but also because this structure is relatively easy to prepare. The MFI structure includes silicalite-1 consisting of pure silica and ZSM-5 in which some Si atoms are substituted by Al. The PV performance of MFI-type membranes for recovering ethanol from aqueous solution is tabulated in Table 4. On account of the presence of hydrophilic silanol groups in structural defects and intercrystalline boundaries in the chemical structure of hydrophobic zeolite membranes, their intrinsic hydrophobicity is not so high as the hydrophilicity of the hydrophilic zeolite membranes [184, 185], hydrophilic A-type zeolite membranes in particular. As a result, their separation performance in the ethanol removal from water is much worse than that of hydrophilic ones in the dehydration of ethanol [178]. As for this, considerable researches have been conducted to enhance the separation performance of hydrophobic zeolite membranes through developing fabrication methods [186–188], porous supports [47, 53, 189], membrane post-treatment [190], etc.

**Table 4:** Ethanol–water separation performance of MFI membranes

Active layer	Support	Feed concentration (wt. %)	Feed temperature (°C)	Separation factor	Flux (g·m^−2^·h^−1^)	References
MFI	*α*-Alumina tube	5	60	76	1050	[52]
MFI	*α*-Alumina disc	10	110	5.2	51,600	[229]
MFI	*α*-Alumina disc	10	60	4.8	8500	[230]
Silicalite-1	Silica tube	10	50	120	3160	[59]
Silicalite-1	Mullite tube	5	60	106	930	[53]
Silicalite-1	POTS-modified titania disk	5	75	103	2560	[209]
Silicalite-1	Silica tube	3	60	95	580	[188]
Silicalite-1	*α*-Alumina tube	5	60	89	1810	[60]
Silicalite-1	*α*-Alumina tube	5	60	85	1220	[53]
Silicalite-1	Silica tube	3	60	84	560	[186]
Silicalite-1	Silica tube	3	80	72	1200	[187]
Silicalite-1	Mullite tube	10	60	72	2550	[198]
Silicalite-1	Mullite tube	5	60	72	1410	[60]
Silicalite-1	Stainless steel	4	30	70	330	[189]
Silicalite-1	*α*-Alumina hollow fiber	3	60	66	2900	[195]
Silicalite-1	Mullite tube	5	60	66	1910	[61]
Silicalite-1	*α*-Alumina tube	5	60	62	1820	[199]
Silicalite-1	Stainless steel tube	5	30	62	225	[231]
Silicalite-1	Mullite tube	5	60	60	2850	[196]
Silicalite-1	Stainless steel disc	4	60	58	760	[206]
Silicalite-1	*α*-Alumina capillary	5	65	54	1500	[194]
Silicalite-1	*α*-Alumina hollow fiber	5	60	51	7600	[197]
Silicalite-1	Stainless steel	4	30	51	150	[232]
Silicalite-1	YSZ hollow fiber	5	60	47	7400	[54]
Silicalite-1	*α*-Alumina tube	5	60	45	1380	[193]
Silicalite-1	*α*-Alumina tube	5	25	43	200	[207]
Silicalite-1	Stainless steel disc	4	30	42	540	[233]
Silicalite-1	Stainless steel disk	5	30	41	0.1–0.5	[234]
Silicalite-1	*α*-Alumina	16.1	75	40	1100	[235]
Silicalite-1	Stainless steel tube	5	60	35	3670	[60]
Silicalite-1	*α*-Alumina tube	3	80	33	350	[187]
Silicalite-1	Stainless steel	10	30	31	100	[236]
Silicalite-1	Mullite tube	10	60	20.2	694	[237]
Silicalite-1	Stainless steel net	5	25	19.6	11,500	[238]
Silicalite-1	Stainless steel tube	5	25	10	70	[212]
Silicalite-1	*α*-alumina tube	9.4	70	1.3	2100	[239]
*b*-Oriented silicalite-1	Silica disc (quartz + Stöber)	5	60	85	2100	[191]
*c*-Oriented silicalite-1	Alumina hollow fiber	5	60	58	9800	[210]
*c*-/*h0h*-out-of-plane-oriented silicalite-1	Stainless steel tube	5	75	43	1200	[211]
ZSM-5	Titania tube coated with three intermediate ceramic titania layers	5.1	40	97.7	810	[47]
ZSM-5	POTS-modified *α*-alumina disk	5	75	58	1360	[209]
Silicalite-1 (coated with silicone rubber)	Stainless steel	4	30	125	140	[232]
Silicalite-1 (modified with silane C_18_H_37_SiCl_3_)	Stainless steel disc	4	50	45	133	[220]
Silicalite-1 (coated with silicone rubber)	Stainless steel	10	30	43	230	[236]
Silicalite-1 (modified with dopamine)	YSZ hollow fiber	5	60	44	2600	[190]
B-ZSM-5	*α*-Alumina tube	5	60	55	2600	[217]
B-ZSM-5	Alumina-coated SiC multi-channel monolith	5	60	31	160	[216]
B-ZSM-5	*α*-Alumina discs	5	55	13.9	1110	[240]
B-ZSM-5	Stainless steel tube	5	25	2.1	50	[212]
Ge-ZSM-5	Stainless steel tube	5	25	29	110	[212]
Ge-ZSM-5	Stainless steel tube	5	30	47	220	[214]
Al-ZSM-5	Stainless steel tube	5	25	9.4	60	[212]
Fe-ZSM-5	Stainless steel tube	5	25	3.4	60	[212]
Ti-silicalite-1	Mullite tube	5	60	127	770	[218]
Ti-silicalite-1	*α*-Alumina capillary	5	65	58.4	2200	[241]
Zr-silicalite-1	Mullite tube	5	60	73	1010	[219]

The fabrication methods of membranes for the PV application in the recovery of ethanol were tabulated by Elyassi et al., summarizing the works published from 1994 to 2015 [191]. As reviewed [53, 187, 192], zeolite membranes are typically synthesized by two methods, namely, direct in situ crystallization and secondary (seeded) growth [60, 193]. The in situ method is that a porous support is immersed into a precursor growth sol or gel that placed in an autoclave, and the zeolite membrane is directly crystallized on the support. Comparatively, this method is simple and easy to implement, and therefore, is suitable for preparation in a large scale. Nevertheless, it is limited by the fact that a dense and compact zeolite membrane is challenging because of nucleating and growing in the bulk solution simultaneously. Moreover, the thickness and orientation of the crystal layer can hardly be controlled [53].

The secondary growth method, also referred to as two-step crystallization, involves the crystal nucleation and growth steps which were totally separated and carried out independently. In this method, zeolite crystals (typically nanocrystals) with uniform size were pre-seeded onto the supports prior to the secondary hydrothermal growth, which is conducive to control the membrane microstructure and orientation of crystal growth, resulting in a dense zeolite membrane layer and a higher reproducibility [194]. Unfortunately, this method is relatively complicated, and cannot be available for large-scale application. The quality of the membranes synthesized by this method is extremely correlated with the seed layer. Therefore, the seeding is a crucial procedure to obtain a continuous and uniform seed layer, and thus a homogeneous defect-free zeolite membrane. At present, many seeding techniques such as dip coating [54, 60, 61, 194–197], rub coating (with either wetting slurry [53, 198] or dry seeds [191, 199]), and vacuum seeding [193] have been reported.

The dip coating is the simplest and most broadly available technique. Shu et al. utilized the dip-coating method to fabricate MFI membranes on yttria-stabilized zirconia (YSZ) hollow-fiber supports that displayed an extremely high permeation flux of 7400 g·m^−2^·h^−1^ coupled with a reasonable high separation factor of 47 at 60 °C [54]. Wang et al. prepared silicalite-1 membranes on *α*-alumina hollow fibers by means of the dip-coating method. Their membranes possessed both the high permeance (permeation fluxes of 2900 and 5400 g·m^−2^·h^−1^) and the high selectivity (separation factor of 66 and 54) for the pervaporative recovery of ethanol from aqueous solutions (3 wt. % at 60 °C and 5 wt. % at 75 °C) [195]. Besides, Kita and co-workers reported intergrown silicalite membranes on tubular mullite supports synthesized in ultradilute precursor solutions by this facile method. The observation showed that the silicalite membranes, prepared with either the classical templates (tetrapropylammonium bromide (TPABr) and tetrapropylammonium hydroxide (TPAOH)) or the inexpensive pure TPABr templates, exhibited the high-PV performance. The highest permeation flux and separation factor were 1910 g·m^−2^·h^−1^ and 66 for the former case, and 1770 g·m^−2^·h^−1^ and 63 for the latter case, respectively, towards a 5 wt. % ethanol/water feed at 60 °C [61]. Yet, despite its advantages, this method is usually applicable to the synthesis of zeolite membranes on smooth and uniform supports with small pore sizes (generally less than 1 μm). It is possibly because, as noted by Wang et al., seeding on large-pore (over 1 μm) supports would probably lead to a low membrane reproducibility [200]. However, this is bound to make an augment in not only the capital costs of supports but also the support transport resistance and sometimes limiting for PV separations.

In the rub-coating seeding method, supports are simply rubbed with zeolite particles. Kita’s group demonstrated that high-PV performance silicalite-1 membranes with high reproducibility could be achievable by this method on tubular mullite and alumina supports [53, 198]. The resultant membranes offered an incredibly high separation factor up to 106 with a moderate permeation flux of 930 g·m^−2^·h^−1^ for ethanol/water separation (at 50 °C). Precious few works reported in the literature have, so far, been superior to this one. They also identified that the rub coating is a straightforward, effective, and reproducible seeding method [53]. However, the main vulnerability of this method is that the preparation process of zeolite seed particles is too complicated.

More recently, Ueno et al. have reported a novel seeding method to build the silicalite-1 selective layer. A zeolite-dispersed polymer film was applied to deposit a uniform and continuous seed film on a porous tubular silica support. The superiority of this seeding approach was not subject to the constraints of the support pore size and seed crystal size. It would thus make highly reproducible zeolite membranes with high separation performance even be scaled up. A maximal separation factor of 120 with an associated permeation flux of 3160 g·m^−2^·h^−1^ was achieved at the operating temperature of 50 °C for a 10 wt. % ethanol/water mixture. Overall, that is the best MFI membrane performance reported to date for the pervaporative removal of ethanol. In addition, the authors stated that the polymer film-seeding method, given the simplicity and effectiveness, had immense potential in seeding other particles except for zeolite [59].

To break through the limitations of pore sizes and capital costs of supports, Wang and co-workers have worked on the preparation of zeolite membranes on inexpensive and defective large-pore supports. They developed a novel seeding method known as wetting–rubbing which involved wetting and rubbing steps (corresponding to dip coating supports with wetting agents, and then rub coating with dry crystal seeds, respectively). They found that in the case of *n*-butanol served as the wetting agent, high-performance membranes (separation factor of 62 and permeation flux of 1820 g·m^−2^·h^−1^) were attained on tubular *α*-alumina supports (1–3 μm in average pore size) [199]. On the same operating condition, the performance was as much as 30% higher than that of membranes on similar supports by the vacuum-seeding method [193]. The method of organic solvent wetting followed by rubbing provides an effective strategy to reproducibly fabricate zeolite membranes on defective low-cost supports, which is industrially attractive.

Based on the above, the membrane PV performance is dependent on not only the membrane synthesis conditions (e.g., time, temperature, and gel composition) and operating conditions (e.g., time, temperature, and composition of the mixture to be separated), but the supports as well. For clarity, the supports of MFI membranes reported in the literature are also assembled in Table 4.

Giaya et al. demonstrated that the hydrophobicity of the dealuminated Y (DAY) zeolite enhanced with the augment of the Si/Al ratio in the zeolite framework [201]. The dependence of hydrophobicity on the Si/Al ratio was also presented for other zeolite structures, such as zeolite X, Y, BEA, and mordenite [202–205]. Kovo proved that A- and X-type zeolite membranes were both selective towards water because of their hydrophilicity occasioned by the high Al content, whereas the ZSM-5 membrane showed selectivity towards ethanol due to its hydrophobicity associated with the low Al content [204]. As compared with the ZSM-5, silicalite-1 is inherently more hydrophobic attributed to the absence of Al atom in its framework. Accordingly, silicalite-1 is the most commonly-used membrane material so far for the separation of ethanol from water, as shown in Table 4. Besides, if an alumina support is employed, silicalite-1 membranes would show a reduction of hydrophobicity owing to the dissolution of alumina during hydrothermal synthesis [186, 206]. From these views, it appears that Al-free supports, including stainless steel, all-silica, titania, and YSZ, are expected to be the most promising supports for preparing pure-silica MFI membranes [190]. For instance, Chen et al. prepared a silicalite-1 membrane on a novel porous silica tube support instead of an alumina support. It exhibited high-PV performance with a permeation flux of 560 g·m^−2^·h^−1^ and a separation factor of 84 with a feed of 3 wt. % ethanol/water solution at 60 °C. In addition, they indicated that high-performance silicalite-1 membranes were easier to be prepared with the silica supports than others [186]. Interestingly, even less hydrophobic ZSM-5 membranes, when being deposited on the top of the support which was composed of a titania tube and three depositing intermediate ceramic titania layers, were found to exhibit exceptionally good separation performance (permeation flux: 810–11,300 g·m^−2^·h^−1^ and separation factor: 49.9–97.7) for ethanol enrichment from round 5 wt. % of aqueous solutions over a temperature range from 40 to 120 °C [47].

In addition to the above-mentioned common fabrication methods, other approaches have also been proposed in recent years. For example, a two-step in situ hydrothermal synthesis approach was applied to fabricate silicalite-1 membranes. Before each hydrothermal synthesis, a “solution-filling (SF)” method, filling porous supports with a viscous mixture composed of water and glycerol, was also performed to protect the supports from invading synthesis solutions. It was found that the permeation flux with SF pre-treatment was approximately twice that without SF pre-treatment, and the silicalite-1 membranes supported on silica tubes displayed high-reproducibility and consistently high separation performance (permeation flux: 870 g·m^−2^·h^−1^ and separation factor: 69) for ethanol extraction from water at 60 °C [187].

Another interesting work by Soydas and co-workers revealed a method to synthesize MFI membranes by recirculating synthesis solutions through tubular *α*-alumina supports. Their membranes had a 200 g·m^−2^·h^−1^ permeation flux and a 43 separation factor in 5 wt. % ethanol feed concentration when operating at 25 °C. The authors also noted that the membrane formed in the recirculating flow system showed a higher ethanol/water separation factor than that prepared in the conventional batch system, despite the similarity in permeation flux. They attributed this to the utilization of recirculating flow synthesis system which provided a more uniform fabrication environment around the supports [207].

Meanwhile, microwave-assisted hydrothermal synthesis was pioneered by Sebastian et al. They fabricated silicalite-1 membranes on ceramic *α*-alumina capillaries by seeded secondary growth under microwave irradiation. By integrating the microwave-heating technique, the synthesis duration was greatly shorted down to 2 h from a typical period of 8 h up to 3 days, thereby preventing the dissolution of ceramic alumina supports, enhancing the membrane hydrophobicity, and improving the selectivity for ethanol. However, the high surface to volume ratio of capillaries and the rapid growth assisted by microwave heating tended to form a thinner and probably more defective selective layer, indicating that the membranes were expected to deliver a high permeation flux with a sacrifice in separation factor [194].

More recently, inspired by “like dissolves like” principle, Huang et al. confirmed that hydrophobic ZIF-8 membranes preferred to grow on hydrophobic support surface which conformed to a principle as “like grows like” [208]. In their subsequent work, instead of growing the crystal layer directly onto the supports, they modified the hydrophilic porous *α*-alumina and titania supports with 1H,1H,2H,2H-perfluoroalkyltriethoxysilanes (POTS), thereby converting them into superhydrophobic ones. Then, through in situ hydrothermal synthesis, dense, well-intergrown, and phase-pure MFI zeolite membranes were obtained. As mentioned previously, because of without the alumina dissolution, it was proved that their silicalite-1 membranes on POTS-modified titania disks had an attractive separation factor/permeation flux combination (as high as 103 and 2560 g·m^−2^·h^−1^, respectively), which was almost a doubling of the performance of the ZSM-5 membranes on POTS-modified *α*-alumina disks. They suggested that this principle could be liable to scale-up in the form of tubular configuration. In addition, from the industrial application of view, surface modification could be effective for batch processing of supports, irrespective of length, size, and shape [209].

Different from Kita et al.’s observation that the separation factor for randomly oriented silicalite-1 membranes was higher than that for oriented ones [53], Elyassi et al. developed *b*-oriented silicalite-1 membranes supported on silica discs by gel-free secondary growth, and obtained a very high ethanol/water separation factor of 85 and a sustainable high permeation flux of 2100 g·m^−2^·h^−1^ at 60 °C [191]. Likewise, both *c*-oriented [210] and *c*-/*h0h*-out-of-plane-oriented [211] silicalite-1 membranes showed good separation performance, particularly the permeation flux of the former as high as 9800 g·m^−2^·h^−1^ at 60 °C for a 5 wt. % ethanol/water feed [210]. It is suggested that oriented zeolite membranes appear to be promising and look worthy of further investigation.

Furthermore, metals substituted into the zeolite framework would change its pore size and intrinsic hydrophobicity, thereby affecting diffusion and adsorption properties. Some researchers have attempted to introduce some metallic elements, such as boron (B), germanium (Ge), iron (Fe), titanium (Ti), and zirconium (Zr), into MFI structure for the application of PV in the recovery of ethanol.

Noble et al. incorporated Al, Fe, B, and Ge into the ZSM-5 framework by isomorphous substitution of Si to prepare zeolite membranes on asymmetric stainless steel tubes. The Ge-ZSM-5 membranes had both the highest permeation flux and ethanol separation factor, whereas the other Al, Fe, and B-substituted ZSM-5 membranes all showed lower PV performance than silicalite-1 membranes as PV at the same condition [212–214]. Tetravalent Ge is chemically similar to Si, but the Ge atom is larger than the Si atom, resulting in smaller pores than silicalite-1 [215] In all probability, the change of the zeolite structure influences the hydrophobicity [178]. On the other hand, trivalent metal (Al, Fe, and B) substitution destructs the charge balance of zeolite. The resulting negatively charged zeolite framework involves an increase in the local polarity in the pores, thereby making it hydrophilic. It seems that ZSM-5 zeolites substituted by trivalent metals are not desirable materials for the separation of ethanol from water. In reality, the B-ZSM-5 zeolite membranes on alumina-coated SiC multi-channel monolith supports were prepared by Bowen et al. from a gel with a H_2_O/SiO_2_ ratio of about 22, and exhibited an optimal separation factor of 31 and a permeation flux of 160 g·m^−2^·h^−1^ for a 5 wt.% ethanol feed [216]. Saboor et al. reported that B-ZSM-5 membranes fabricated from clear synthesis solutions (H_2_O/SiO_2_ = 64) on seeded *α*-alumina discs preferentially permeated ethanol from water, showing a relatively high permeation flux of 1110 g·m^−2^·h^−1^ but a low separation factor of 13.9. Recently, Chai and co-workers observed a high permeation flux of 2600 g·m^−2^·h^−1^ and a moderate separation factor of 55 using a B-ZSM-5 membrane synthesized from a dilute solution (H_2_O/SiO_2_ = 600) on seeded inexpensive macroporous *α*-alumina tube [217]. It suggests that the PV performance for B-ZSM-5 membranes is greatly dependent on their preparation conditions. Nevertheless, it remains lower than that of the most silicalite-1 membranes.

On the other hand, Chen et al. incorporated Ti atoms into silicalite-1. They found that the resultant Ti-silicalite-1 membranes had a higher ethanol separation performance than the similar silicalite-1 membranes. For separating 5 wt. % ethanol/water feed at 60 °C, the separation factor and permeation flux reached the highest values, 127 and 770 g·m^−2^·h^−1^, respectively [218]. Notably, the Ti-silicalite-1 membrane is currently the best performing inorganic membrane for ethanol recovery. Later, they continued their work on Zr-substituted silicalite-1 membranes, and observed a high ethanol/water separation factor of 73 with a corresponding permeation flux of 1010 g·m^−2^·h^−1^ [219].

Post-treatment has also exerted a positive impact on membrane performance. Silanization is an effective and simple modification technique to eliminate hydrophilic silanol groups in zeolite structures, enhancing its hydrophobic nature, hence augmenting its membrane performance. For example, Sano and co-workers conducted the silylation of silicalite membranes using silylating agents (octyltrichlorosilane and octadecyltrichlorosilane). It was interesting to find that the separation factors of silylated silicalite membranes were markedly improved to 20–44, which is around fourfold to ninefold higher than that without silylation. Unfortunately, they found that there was a sacrifice in permeation flux, declining noticeably from 843 to 53.4 g·m^−2^·h^−1^ [220, 221]. More recently, Wu et al. opened up a novel method of masking the silanol groups on the zeolite surface. They modified the MFI zeolite membranes with dopamine. After dopamine modification, the initial ethanol separation factor was similar to that of unmodified membranes; whereas the initial permeation flux reduced by about 50%. However, it was proved that the long-term PV stability of the dopamine-modified membranes was improved apparently. Over 180 h of exposure to the feed at 60 °C, the separation factor for unmodified membranes was down to 1 from 43, meanwhile the permeation flux to 500 from 4900 g·m^−2^·h^−1^; but the separation factor and permeation flux for the modified ones maintained almost constant values of 44 and 2600 g·m^−2^·h^−1^, respectively [190].

Besides MFI-type zeolite membranes, other type zeolite membranes have also been investigated for ethanol recovery. Li et al. incorporated B and Al atoms into MEL-type zeolite ZSM-11 framework structure and prepared B and Al-substituted ZSM-11 membranes on porous tubular supports. The separation factors through the Al-ZSM-11 membranes were less than 6 with a wide range of permeation fluxes (210–1800 g·m^−2^·h^−1^); whereas the maximal separation factor through the B-ZSM-11 membranes reached a value of 42, coupled with a permeation flux of 930 g·m^−2^·h^−1^ at 5 wt. % of ethanol in the feed at 60 °C [222]. Similarly, Chai et al. also reported the B-ZSM-11 membranes for this application, and they obtained a similar observation for the PV performance (highest separation factor of 35 with a permeation flux of 1510 g·m^−2^·h^−1^) [179]. Other work on organic/water separation by mesoporous silica MCM-48 membranes was carried out by Kim et al. However, the separation factor for pure MCM-48 membranes was lower than 1. After silylation, the selectivity was facilitated, but the permeation flux descended considerably as a result of water flux sharp decline [223].

Other than zeolite-based membranes, researchers have also attempted to develop new inorganic membranes. For instance, the boehmite–sol-coated membranes were reported by Song et al. for the separation of ethanol/water mixtures. The results, disappointingly, were far from being satisfactory. The separation factor and permeation flux were as low as 1.14 and 31.5 g·m^−2^·h^−1^ for a fivefold coated-membrane, and 1.76 and 12 g·m^−2^·h^−1^ for a tenfold coated-membrane [224]. In addition, MOFs including its subclass ZIFs have been utilized in the fabrication of organophilic PV membranes. Dong and Lin successfully fabricated integrated ZIF-71 membranes on ZnO disk supports by the reactive method, and demonstrated the feasibility of ZIF membranes for PV separation of organic solvent. For PV of a 5 wt. % ethanol/water solution at 25 °C, a similar observation for the separation performance to the pure PDMS membranes was obtained, showing a separation factor of 6.07 and a limited permeation flux of 322 g·m^−2^·h^−1^ [225]. Huang et al. also prepared ZIF-71 membranes on ceramic *α*-alumina hollow-fiber supports with a modified contra-diffusion method. It was observed that the ZIF-71 membranes were of very high integrity and exhibited a very competitive permeation flux of 2601 g·m^−2^·h^−1^, combined with a separation factor of 6.88 as PV at the same temperature and ethanol concentration in feed with Dong’s work. They explained that the inspiring result could be attributable to the low resistance of ceramic hollow-fiber support as well as the thin selective layer (approximate thickness 2.5 µm) [226]. More recently, the Zr-based MOF structure of UiO-66 was made into membranes. The effective membrane thickness was merely 0.5–1 μm. The thinner membranes displayed highly stable PV performance and had elevated permeation fluxes of 1490 g·m^−2^·h^−1^ at 50 °C and 3150 g·m^−2^·h^−1^ at 60 °C, respectively, with separation factors slightly less than 5 in a 10/90 ethanol/water mixture [227]. Despite unique advantages of MOFs, such as high porosity, ordered structures, and tunable chemical functionality, a few MOFs membranes have been investigated into PV to date as a result of their poor chemical stability. In fact, MOFs have mainly been used to fabricated mixed matrix membranes [21, 87]. Anyway, all these studies are significative attempts for designing and fabricating new inorganic membranes, and further research works are expected.

In summary, the separation performance of inorganic membranes typically outperforms those of polymeric ones. As shown in Table 4, the reported ethanol/water separation factors for MFI zeolite-based membranes range from 1.3 to 127, with permeation fluxes ranging from 0.1 to 51,600 g·m^−2^·h^−1^, which is superior to the performance of all polymeric membranes including PDMS, PTMSP and so on. However, inorganic materials have some serious intrinsic drawbacks, such as poor film-forming capability and high brittleness, and hence are very difficult to be made into large-area defect-free membranes, still under studying in the laboratory for ethanol recovery. Moreover, from the perspective of industrial application, several critical issues have to be overcome as yet, such as membrane reproducibility, long-term stability, manufacture cost—10–50 times that of polymeric membranes [228], and even the permeation flux which is generally low and should be improved further. More efforts are needed to make the inorganic membranes industrialized practically in clean bioethanol production.

### Mixed matrix membranes

Mixed matrix membranes (MMMs), whose concept can be traced back to the mid-1980s [242, 243], are also known as hybrid membranes. MMMs are generally fabricated by dispersing inorganic particles (so-called fillers) into continuous polymer matrices. MMMs integrate their strengths, such as high separation performance as well as high stability of inorganic membranes, and easy fabrication as well as low costs of polymeric membranes. MMMs provide a flexible and cost-effective way to overcome the trade-off behavior in polymeric membranes, obtaining high permeation flux and high separation factor simultaneously. MMMs have been considered as a promising and competitive candidate for ethanol recovery by PV. Over the past 3 decades, they have gained increasing interest and become a research focus in membrane separation fields. In addition, the rapid development of MMMs provides a new thought for the design and preparation of new PV membranes.

Based on the literature reports, the most common MMMs for ethanol-selective PV are still based on the PDMS matrix. Its source including components and composition is important in determining the performance of MMMs [244]. Most PDMS used for ethanol-selective MMMs are two-component vinyl-terminated General Electric RTV 615 [76, 77, 82, 245] and hydroxyl-terminated PDMS produced in China [21, 72, 126, 246]. Generally, it seems that vinyl end-capped PDMS MMMs are capable of delivering higher ethanol selectivity. The vinyl-based PDMS involves the hydrosilylation reaction between the vinyl groups of prepolymer (RTV 615 part A) and methyl-hydride groups of silicone copolymer crosslinking agent (RTV 615 part B). This system is highly advantageous to improve the viscosity of the membrane casting suspension, leading to the increase in shear stress to break down particle aggregations which makes the particles more likely to be surrounded individually by polymer chains, thus increasing the maximum practicable particle loadings which results in an increase in selectivity [19]. Other polymer matrices reported include PTMSP [55, 90, 151, 153], PEBA [103, 247], PMPS [97], PIM [20], etc. A list of literature performance of various polymer-based MMMs for ethanol recovery from water is assembled in Table 5.

**Table 5:** Ethanol–water separation performance of MMMs

Polymer membrane material/support	Filler (loading)	Feed concentration (wt. %)	Feed temperature (°C)	Separation factor	Flux (g·m^−2^·h^−1^)	Reference
PDMS/silicalite-1						
PDMS/PEI	Silicalite-1 (77 wt. %)	7	22	59	71	[19]
PDMS	Silicalite (60 wt. %)	5	50	21	~ 105	[76]
PDMS	Silicalite-1 (40 wt. %)	5	50	17.9	~ 60	[73]
PDMS/alumina	Silicalite-1 (30 wt. %)	4	25	16.5	~ 125	[75]
PDMS	Silicalite-1 (30 wt. %)	6	40	14.9	51	[84]
PDMS/PTFE	Silicalite-1 (30 wt. %)	5	50	13	39	[132]
PDMS/PES	Silicalite-1 (2 wt. %)	5	40	10.9	635	[126]
PDMS	Silicalite-1 (30 wt. %)	6	35	~ 10	~ 70^a^	[86]
PDMS/PSF	Silicalite-1 (50 wt. %)	4.8	60	7.5	231	[262]
PDMS (commercial MMM)	Silicalite-1 (50 wt. %)	6	35	~ 7.2	~ 55	[86]
PDMS/PI	Silicalite-1 (15 wt. %)	3	41	4.8	170	[74]
PDMS/modified silicalite-1						
PDMS	VTES-modified silicalite-1 (67 wt. %)	5	50	34.3	176	[80]
PDMS	Acid- and steam-treated silicalite-1 (50 wt. %)	4.4	50	29.3	120	[155]
F-PBZ modified PDMS/PTFE	VTMS-modified silicalite-1 (30 wt. %)	5	50	28.7	207	[132]
PDMS/PVDF	VTES-modified silicalite-1 (67 wt. %)	5	50	26.3	1940	[77]
PDMS/PVDF	Dodecyltrichlorosilane-modified silicalite-1 (50 wt. %)	5	40	19.9	66.3	[81]
PDMS	Silylated nano-sized silicalite-1 (40 wt. %)	6	35	16.4	~ 86	[125]
PDMS/PSF	TMDS-modified silicalite-1 (50 wt. %)	5	60	14.7	400	[78]
PDMS/ZSM-5						
PDMS	ZSM-5 (60 wt. %)	5	50	~ 37	~ 380	[82]
PDMS/PVDF	ZSM-5 (30 wt. %)	5	50	13.7	821	[263]
PDMS/PVDF/polyester nonwoven fabric	ZSM-5 (20 wt. %)	5	40	10.4	~ 210	[257]
PDMS	ZSM-5 (30 wt. %)	6	35	~ 6.3	~ 55^a^	[86]
PDMS	ZSM-5 (CBV 28,014) (60 wt. %)	5	50	39	66	[248]
PDMS	ZSM-5 (CBV-28014) (50 wt. %)	5	50	37	195	[244]
PDMS with a pure PDMS top coat/PVDF	ZSM-5 (CBV-28014) (65 wt. %)	5	50	18	~ 520	[44]
PDMS/PI	ZSM-5 (CBV-3002) (30 wt. %)	3	41	5.5	151	[74]
PDMS/modified ZSM-5						
PDMS/PVDF	HF etched ZSM-5(30 wt. %)	5	50	16.7	134	[83]
PDMS/PVDF/polyester nonwoven fabric	Dodecyltrichlorosilane-modified ZSM-5 (30 wt. %)	5	40	15.8	203	[257]
PDMS/CA	APTS-modified ZSM-5 (20 wt. %)	10	40	14.1	348	[79]
PDMS/ceramic	ZSM-5 first grafted with OTES and then coated with a thin PDMS layer (40 wt. %)	5	40	14	408	[260]
PDMS/other zeolites						
PDMS	Ultrastable zeolite type Y (50 wt. %)	–-	30	16.1	610 L·m^−2^·h^−1^	[92]
PDMS	Hollow spheres with silicalite-1 shell (30 wt. %)	6	40	15.3	72	[84]
PDMS	Zeolite (TZP-9023) (30 wt. %)	10	25	12.5	332	[261]
PDMS(commercial MMM)	Zeolite (–-)	1.5	66	11.1	120	[85]
PDMS	ALPO-5 type zeolite (50 wt. %)	–-	30	5.2	200 L·m^−2^·h^−1^	[92]
PDMS	Zeolite Y (30 wt. %)	6	35	~ 4.5	~ 90^a^	[86]
PDMS/MOFs						
PDMS/PVDF	ZIF-91 (20 wt. %)	5	55	15.8	846	[266]
PDMS/PI	ZIF-67 (20 wt. %)	6	40	15.4	2780	[251]
PDMS/PVDF	RHO-[Zn(eim)_2_] (MAF-6) (15 wt. %)	5	40	14.9	1200	[250]
PDMS/PVDF	ZIF-L (30 wt. %)	5	40	14.3	~ 570	[95]
PDMS	ZIF-71 (40 wt. %)	2	60	12.5	55,470 barrer	[87]
PDMS/PVDF	Submicrometer-sized ZIF-71 (40 wt. %)	5	50	10.1	~ 1070	[255]
PDMS/PVDF	ZIF-71 (20 wt. %)	5	50	9.9	~ 900	[259]
PDMS	ZIF-71-coated mesoporous silica core–shell sphere (20 wt. %)	6	40	13	1000	[96]
PDMS/PSF	MIL-53 (40 wt. %)	5	70	11.1	5467	[21]
PDMS	ZIF-8-coated mesoporous silica core–shell sphere (20 wt. %)	6	40	15	720	[96]
PDMS	ZIF-8 (5 wt. %)	5	60	9.9	1229	[93]
PDMS/modified MOFs						
PDMS/PVDF	Dodecylamine-modified ZIF-90 (2.5 wt. %)	5	60	15.1	99.5	[252]
PDMS/PSF	TMDS-modified MCM-41@ZIF-8 (5 wt. %)	5	60	9.5	1846	[267]
PDMS/other fillers						
PDMS/nonwoven fabric	Nanosilica (5 wt. %)	5	60	30.1	114	[264]
PDMS/PSF	Fumed silica (5 wt. %)	5	60	12.5	807	[93]
PDMS/PVDF	Fumed silica (20 wt. %)	4.8	60	~ 6.4	~ 1000	[268]
PDMS (commercial MMM)	Silica (30 wt. %)	8	25	~ 9	85	[130]
PDMS	POSS (5 wt. %)	10	50	17.7	536	[269]
PDMS/PVDF	Octa[(trimethoxysilyl)ethyl]-POSS (7.5 wt. %)	10	40	16.4	253	[246]
PDMS	PSS-2 (20 wt. %)	6	40	13	710	[270]
PDMS/PA	PZSNT (10 wt. %)	10	40	10	476	[265]
PDMS/PA	Carbon black (3 wt. %)	13.7	30	9	~ 178	[99]
PDMS	CNT (10 wt. %)	8	60	8.2	129	[98]
PDMS	[Cu^II^_2_(bza)_4_(pyz)]_n_ (3 wt. %)	5	25	6.2	47	[89]
PDMS/PVDF	PAF-11 (2 wt. %)	10	28	3.85	1480	[254]
Other polymers/fillers						
PTMSP/PVDF	Hexamethyldisilazane-treated silica (CabO-Sil TS 530) (25 wt. %)	5	50	18.3	9500	[55]
PTMSP	Hexamethyldisilazane-treated silica (CabO-Sil TS 530) (50 wt. %)	10	50	15.3	400	[151]
PTMSP	PAF-1 (10 wt. %)	10	40	12.7	247	[153]
PTMSP	p-DCX (10 wt. %)	10	40	13.7	341	[153]
PTMSP/PVDF	Silica (50 wt. %)	10	50	12	3500	[151]
PEBA 2533	POSS (2 wt. %)	5	65	5.7	427	[103]
PEBA/PAN	Silicalite (2 wt. %)	5	40	3.6	833	[247]
Poly(styrene-co-butylacrylate) copolymer	Nano clay (Cloisite 15A) (2 wt. %)	5	30	26.4	340	[258]
PMPS	ZIF-8 (9.1 wt. %)	1	80	~ 12	~ 6600 barrer	[97]
PIM	Reduced octylamine-functionalized graphene oxide (0.1 wt. %)	5	65	5.1	885	[20]
PVDF/PVDF (dual-layer hollow fiber)	Nanosilica (20 wt. %)	5	50	29	1100	[91]
PA6	CNT (0.5 wt. %)	20	22	4.7	–-	[175]

^a^The fluxes are normalized to a membrane thickness of 100 μm.

1 barrer = 1 × 10^−10^ cm^3^ (STP) cm cm^−2^·s^−1^·cmHg^−1^.

*PEI* polyetherimide, *PTFE* polytetrafluoroethylene, *PES* polyethersulfone, *PSF* polysulfone, *PI* polyimide, VTES vinyltriethoxysilane, *F-PBZ* fluorinated polybenzoxazine, *VTMS* vinyltrimethoxysilane, *PVDF* polyvinylidene fluoride, *TMDS* 1,1,3,3-tetramethyldisilazane, *CA* cellulose acetate, *APTS* 3-aminopropyltriethoxysilane, OTES noctyltriethoxysilane, *PA* polyamide, *POSS* polyhedral oligomeric silsesquioxane, *PSS-2* poly-oligosiloxysilicone, *PZSNT* polyphosphazene nanotube, *CNT* carbon nanotube, *PAF* porous aromatic framework, *p-DCX* polydichloroxylene, *PAN* polyacrylonitrile, *PEBA* poly(ether block amide), *PMPS* polymethylphenylsiloxane, PIM polymers of intrinsic microporosity

After dozens of years of development, a good number of fillers have been incorporated into polymer matrices for ethanol recovery. The most commonly used fillers are zeolites including silicalite-1 [19, 73, 75, 245] and ZSM-5 [82, 92, 248, 249]. Other fillers reported are MOFs [97, 250] and its subclass ZIFs [87, 251, 252], fumed silica [55, 88, 93], carbon nanotubes [98, 175], carbon blacks [99, 100], POSSs [103, 246], etc. Taking into account the filler particles, filler type, particle size, and particle dispersion status in MMMs are crucial for the PV separation properties. To form desirable thin, smooth, homogeneous, and high-performing MMMs, works could focus on following approaches: (a) achieving a uniform dispersion of filler particles; (b) eliminating undesirable organic–inorganic interfacial defects; (c) increasing filler loadings (but do prevent excessive loading); and (d) optimizing particle sizes.

The uniform dispersion of inorganic particles in the polymer matrix is one of the greatest challenges. Usually, most of inorganic particles are physically mixed with polymer matrix. Since the differences in physicochemical properties between them, the particles have a tendency to agglomerate, and the agglomeration is more severe for nanoparticles on account of their higher surface energy [253]. The particle aggregation may induce the formation of nonideal-defects (e.g., nonselective voids [254]), which often leads to a decrease of selectivity. To restrict the agglomeration and enhance the homogeneous distribution of particles, various methods have been developed, such as sonication [73, 75, 97, 248], optimization of particle size [244, 255, 256], surface chemistry modification of particles [76, 80, 83, 257], and pre-polymerization of polymer [19, 77].

It is often difficult to achieve a homogeneous dispersion only by simple mechanical stirring [258]. In practice, the fillers are usually dispersed in a polymer dope solution (or pre-dispersed in a solvent) with thoroughly stirring and ultrasonication [21, 78, 81]. The sonication process can be carried out using an ultrasonic bath or a probe-type sonicator. The probe-type sonicator has a high localized intensity compared to the tank-type and hence bigger crushing force, resulting in finer particles and more uniform particle dispersion. Vane et al. confirmed that sonication was effective to disrupt particle agglomerations. Due to defects triggered by substantial particle agglomerates for membranes fabricated without sonication, more instances of poor separation performance or failure appeared. Their study also verified that the effectiveness of probe-type sonicators at particle dispersal was obviously better than that of ultrasonic baths [244]. Zhang and co-workers proposed in situ ultrasonic strengthening assembly in which sonication was applied not only during mixing but during assembly to avoid secondary aggregation on supporting surface. It was found that the dispersion of nanofillers (particle size of 7–40 nm for silica nanoparticles and about 100 nm for ZIF-8 nanoparticles) in PDMS was markedly improved [93].

Minimizing particle size is theoretically possible to make much thinner defect-free membranes, which is advantageous to improve the membrane separation performance. However, the tendency of particles to agglomerate which is detrimental to the performance has an inverse relationship to the particle size [244]. Besides, the impact of agglomeration caused by particle loadings should be taken into consideration [259]. Li et al. embedded micron- and nano-sized ZIF-71 particles into PDMS to prepare MMMs. They demonstrated that the effect of particle size in MMMs was remarkable. Micron-sized ZIF-71 particles were more prone to create defects in MMMs, especially at higher loadings. In contrast, nano-sized ZIF-71 would be more preferable for preparing MMMs with higher loadings (more than 20 wt. %) but fewer defects [259]. Similar to Li et al.’s observation, Wee et al. found that submicrometer-sized ZIF-71 particles were evenly dispersed in PDMS membranes with no interfacial voids nor ZIF-71 aggregates, while MMMs containing micrometer-sized ZIF-71 were rough and uneven with large interstitial voids. Correspondingly, the submicrometer-sized (290 nm) ZIF-71 filled PDMS MMMs delivered a maximal separation factor of 10.1 which outperformed that of 7.6 for the ones filled with micrometer-sized (1–2 µm) ZIF-71 [255]. The study carried out by Yin et al., however, showed that micron-sized (1 µm) ZIF-71 particles had less agglomeration and better dispersion in PDMS MMMs than smaller ZIF-71 particles (~ 150 and 500 nm). The PV performance of resultant MMMs containing micron-sized ZIF-71 outperformed those prepared with smaller particles in terms of both selectivity and permeability due to the less nonselective and tortuous pathways through the MMMs that provided less mass transfer resistance [256]. Similarly, Vane et al. also observed that membranes made with micron-sized (2.4 µm) commercial ZSM-5 (Zeolyst CBV-28014) exhibited higher ethanol separation performance than those made with submicrometer-sized (0.35 and 0.70 μm) silicalite-1 because of their tendency to form silicalite-1 aggregates, especially for particle loadings of 50 wt. % or higher [244].

Pre-polymerization or pre-crosslinking of polymer has also been developed to assist fillers to disperse homogenously in polymer network. It not only prevented fillers from agglomeration or sedimentation in casting suspensions, but also inhibited infiltration of polymer chains into filler pores. Jia et al. performed pre-crosslinking to partially polymerize two components of PDMS with silicalite-1 fillers present. Such a pre-crosslinking process increased viscosity of casting suspensions and improved their stabilization. The resultant membranes displayed a maximum separation factor of 59 at 22 °C. This performance is the highest of any MMMs reported so far. It, however, combined with a particularly low permeation flux of 71 g·m^−2^·h^−1^ [19]. Zhou et al. employed the pre-polymerization of PDMS polymer network to facilitate the dispersion of silicalite-1. It was demonstrated that 67 wt. % loading modified silicalite-1 maintained a uniform dispersal in PDMS solution. A 5-μm-thick modified silicalite-1 filled PDMS membrane had a high permeation flux of 5520 g·m^−2^·h^−1^ with a separation factor of 15.5 for the pervaporative recovery of ethanol from aqueous solutions (5 wt. %) at 50 °C [77]. Instead of adding fillers into polymer solutions before pre-crosslinking as Jia et al. and Zhou et al. did, Vankelecom et al. incorporated carbon black particles after pre-crosslinking of PDMS to avoid the polymer chains from infiltrating into the pores of carbon blacks [100]. However, when the viscosity of membrane solutions was too high after pre-polymerization, it would render membrane fabrication more difficult because of shorter working time available for casting [77, 244].

Incompatibility between fillers and polymer matrix is another critical issue. To enhance their compatibility, a variety of approaches have been proposed and demonstrated: (i) surface chemistry modification of inorganic particles with coupling agents [77, 80, 132, 257]; (ii) surface coating of inorganic particles with a thin polymer layer [260]; and (iii) employment of hybrid particles (i.e., MOFs [21, 87, 250, 251] and POSS [103, 246]).

Silylation is the most widely used surface modification method of inorganic particles to ameliorate the interfacial compatibility with polymeric matrix as well as to facilitate the particle dispersion. Several groups conducted silylation of zeolites by employing silane-coupling agents to prepare modified zeolite/PDMS MMMs [78–81, 257, 260]. The silane chains immobilized onto the zeolites through hydrolysis and condensation reaction could entangle with PDMS chains [80, 260], which offered good interaction between the zeolite particles and the PDMS matrix. Moreover, vinyl silanes, such as vinyltriethoxysilane (VTES) [80] and vinyltrimethoxysilane (VTMS) [132], were the representative of silane-coupling agents since strong chemical bonds were formed by the reaction of their terminal vinyl groups with Si–H groups on the short cross-linker (RTV 615 B). That intuitively suppressed the formation of nonideal micro-voids at the particle–polymer interface and mitigated membrane swelling [76]. Zhuang et al. modified silicalite-1 particles with four alkoxysilanes (VTES, ethyltriethoxysilane, octyltriethoxysilane, and octadecyltriethoxysilane) and investigated their effect on the performance of PDMS-based MMMs. It was confirmed that the VTES PDMS membranes embedded with modified silicalite-1 displayed the best ethanol selectivity as a consequence of the best compatibility with PDMS just because of the chemical linking between VTES-modified silicalite-1 and PDMS. The resulting MMMs coupled with VTES at 67 wt. % silicalite-1 loading had a separation factor higher than 34.3, while the unmodified membranes at 60 wt. % silicalite-1 loading had a separation factor of 23 [80]. Similar results were observed by Yi et al., and they found that silylation modification of silicalite-1 with VTES rose the maximum loading to 67 from 60 wt. %, accompanied with an obvious increment in separation factor from 21 to 32 [76].

The increase of particle loadings in MMMs generally has a positive effect on separation performance in terms of separation factor, while permeation flux may increase simultaneously [75, 251, 261] or reduce due to intrinsic trade-off effect [132, 260]. However, excessive addition would definitely lead to particle agglomeration and formation of nonselective pores and defects, thus deteriorating the performance. Overall, increasing filler loadings is based on good dispersion and good compatibility. Although the highest zeolite particle loading in ethanol-selective MMMs could reach up to 77 wt. % [19], the optimal loadings of zeolites reported are usually limited to 50 wt. % [92, 244, 262] or less [73, 86, 263]. After the silylation modification of zeolites, the maximum loadings have been obtained to be as high as 67 wt. % [76, 77, 80]. MOFs, developed rapidly in recent years, have garnered extensive attention for their porous structure and good compatibility with polymer matrix. They are introduced into matrix ranging in 15–40 wt. % [87, 95, 250, 251]. The optimal silica loadings range from 5 [93, 264] to 30 wt. % [130]. A higher amount of silica (50 wt. %) was doped into PTMSP by Claes et al. via surface hydrophobic treatment [151]. Other particle loadings are generally no more than 10 wt. % [98, 103, 246, 265], even lower than 0.5 wt. % [20, 175].

From the summary of pervaporative separation data for MMMs listed in Table 5, the ethanol/water separation factors ranging from 3.6 to 59 can be observed. This range is between those of pure polymeric membranes and pure inorganic membranes. On the other hand, the permeation fluxes vary over a fairly wide range, from 39 to 9500 g·m^−2^·h^−1^, which is still dependent on the polymers used (continuous phase) to a larger extent. For representative zeolite/PDMS systems, the ethanol/water separation factors are much higher than that of pure PDMS, while the permeation fluxes are still limited within 1000 g·m^−2^·h^−1^. For instance, Zhuang et al. embedded VTES-modified silicalite-1 into PDMS, and the resultant MMMs with 67 wt. % modified silicalite-1 loading exhibited a high separation factor of 34.3 in combination with a permeation flux of 176 g·m^−2^·h^−1^ at 50 °C for an ethanol/water mixture at 5 wt. % feed concentration [80]. In comparison, the doping of MOFs in MMMs is more beneficial to acquire high permeation flux. Zhang et al. incorporated materials institute Lavoisier-53 (MIL-53) particles into PDMS to form MMMs. They proved that the permeation flux showed a considerable increase from 1667 g·m^−2^·h^−1^ for neat PDMS dense membranes to 5467 g·m^−2^·h^−1^ for PDMS MMMs containing 40 wt. % MIL-53, with a slight increase in separation factor from 7.6 to 11.1. The gain in permeation flux was largely accounted for the water-repellency surface and ethanol-affinity channels of MIL-53. When the MIL-53 content exceeded 40 wt. %, a trade-off behavior between permeation flux and separation factor was observed. Despite the monotonous increase in permeation flux, the separation factor declined, which was mainly attributed to the change of MMM hydrophobicity as well as the formation of defects due to severe particle agglomeration [21]. Another work from Khan and co-workers confirmed that the ZIF-67/PDMS membranes with 20 wt. % of loading showed a simultaneous increment in both permeation flux and separation factor, exhibiting a good anti-trade-off phenomenon. Their values were up to 2780 g·m^−2^·h^−1^ and 15.4—triple and double that of pristine PDMS membranes, respectively [251].

In summary, the separation performance of MMMs for ethanol recovery is generally superior to that of polymeric membranes but worse than that of inorganic membranes alone. Moreover, some MMMs still display a trade-off relationship in separation performance. Till now, only a minority of MMMs have been commercialized, such as silicalite-1/PDMS membranes marketed by Sulzer Chemtech [86]. However, MMMs have their comprehensive advantages, such as desirable mechanical and long-term operation stability, anti-swelling property, economical processability, and easy to large-scale production. They are a promising strategy for ethanol recovery from the bulk feed by PV. Further efforts should be devoted to design new nanoscale filler particles with superhydrophobic pores as well as excellent interfacial compatibility with polymers and explore new preparation processes, breaking the trade-off effect, thus obtaining desirable competitive MMMs with judiciously higher permeation flux and separation factor.

## Conclusions and perspectives

This review summarizes the research progresses on ethanol-selective pervaporative membranes from the perspectives of transport mechanisms, fabrication methods, and membrane materials. The facilitated transport mechanism and solution–diffusion mechanism have been mentioned to understand transport and separation fundamentals so as to better guide membrane design, and therefore, govern membrane performance. The membrane materials (polymeric, inorganic, and hybrid materials) with various modification approaches and the fabrication methods including solution casting and physical blending to prepare PV membranes have been overviewed. As reviewed here, although some encouraging results have been achieved in recovering ethanol from aqueous solution via PV after many decades of extensive research worldwide, it is still rather far from industrial applications because of the lack of industrially practical membrane modules. Therefore, further research is required to explore high-performing, stable, and economic ethanol-selective membranes for large applications. Advances in the following aspects have the potential to impel the development and deployment of PV membranes.

Developing new high-performance membrane materials is an effective strategy. Fabricating membranes with organic–inorganic nanohybrids is probably the most efficient approach to improve the performance stability and to break the permeability-selectivity trade-off at this stage. Future research in this area may focus on the exploration of new nanometer-sized inorganic particles (or hybrid particles) with ultra-hydrophobic pores and extremely high compatibility with polymers. In addition, intelligent materials may be a prospective membrane material for ethanol recovery in the future. By adjusting the intensity of external light, electric field, magnetic field, etc., the microstructure of membranes could be regulated, and therefore, improving membrane recovery performance. In the meantime of exploring new membrane materials, the modification of existing ones may serve as a promising alternative method.

Besides, exploring novel membrane fabrication processes is another strategy. Efforts should be focused on preparing a large-surface-area, defect-free, and ultrathin selective skin layer. In situ synthesis and layer-by-layer self-assembly method may be further extended. Meanwhile, establishing a hierarchical membrane configuration can also be considered. Constructing a thin protective layer on top of the selective layer may be a viable way to not only suppress the swelling of the selective layer but also prevent or mitigate membrane fouling. In practice, the development of manufacturing methods for environmentally friendly, high-efficient, and economic synthetic membranes is encouraged. More attention should be paid to biomimetic methods such as bioinspired mineralization in which the dispersed particles in situ generate from their precursors under a mild condition and have a fine dispersion. Innovative methods to manipulate the microstructure of organic–inorganic nanohybrid materials for forming membranes with high-loading and uniformly dispersed filler particles as well as super-hydrophobic surfaces are expected to be developed actively. Other constructive combination between the polymer and inorganic particles, rather than direct dispersion, should be tried. In addition, much effort should be carried out to promote the reform of the existing fabrication procedures. For example, optimizing post-modification processes could be employed to introduce active groups on the membrane surface to increase the affinity of ethanol toward it.

From the standpoint of industrial application, ethanol removal from fermentation broths via PV is still rather limited. The current research status is that most of the PV experiments reported have been conducted at laboratory scales and performed with simple and ideal binary aqueous solutions, thus resulting in a lack of understanding on membrane performance in real fermentation broths. Long-term trial evaluation of membranes is preferable to be implemented at actual industrial conditions where PV integrates with fermentor. In addition, more efforts should be focused on enhancing the long-term operation stability of membranes including selectivity, chemical and temperature resistance, and robustness, in particular polymeric and mixed matrix membranes. Future studies on ethanol perm-selective PV membranes should be positioned toward increasing membrane permeability and maintaining a satisfactory selectivity so as to reduce required membrane area.

Overall, exploring the possibility and limitation of the separation performance of PV membranes for ethanol extraction is a long-standing topic. Collectively, the quest is to break the trade-off between membrane permeability and selectivity. Based on the facilitated transport mechanism, further exploration of ethanol-selective membranes may focus on constructing a well-designed microstructure, providing active sites for facilitating the fast transport of ethanol molecules, hence achieving both high selectivity and permeability simultaneously. Finally, it is expected that more and more successful research could be realized into commercial products and this separation process will be deployed in industrial practices in the near future.

## Data Availability

All data generated or analyzed during this study are included in this published article.
